# FDX2, an iron-sulfur cluster assembly factor, is essential to prevent cellular senescence, apoptosis or ferroptosis of ovarian cancer cells

**DOI:** 10.1016/j.jbc.2024.107678

**Published:** 2024-08-14

**Authors:** Shuko Miyahara, Mai Ohuchi, Miyuki Nomura, Eifumi Hashimoto, Tomoyoshi Soga, Rintaro Saito, Kayoko Hayashi, Taku Sato, Masatoshi Saito, Yoji Yamashita, Muneaki Shimada, Nobuo Yaegashi, Hidekazu Yamada, Nobuhiro Tanuma

**Affiliations:** 1 Division of Cancer Chemotherapy, Miyagi Cancer Center Research Institute, Natori, Japan; 2 Department of Biochemical Oncology, Tohoku University Graduate School of Medicine, Sendai, Japan; 3 Department of Obstetrics and Gynecology, Tohoku University Graduate School of Medicine, Sendai, Japan; 4 Institute for Advanced Biosciences, Keio University, Tsuruoka, Japan

**Keywords:** iron-sulfur protein, DNA damage response, p53, cancer biology, cellular senescence, iron metabolism, cell death, ovarian cancer, gene knockout, tumor metabolism, reactive oxygen species (ROS), redox regulation

## Abstract

Recent studies reveal that biosynthesis of iron-sulfur clusters (Fe-Ss) is essential for cell proliferation, including that of cancer cells. Nonetheless, it remains unclear how Fe-S biosynthesis functions in cell proliferation/survival. Here, we report that proper Fe-S biosynthesis is essential to prevent cellular senescence, apoptosis, or ferroptosis, depending on cell context. To assess these outcomes in cancer, we developed an ovarian cancer line with conditional KO of FDX2, a component of the core Fe-S assembly complex. FDX2 loss induced global downregulation of Fe-S–containing proteins and Fe^2+^ overload, resulting in DNA damage and p53 pathway activation, and driving the senescence program. p53 deficiency augmented DNA damage responses upon FDX2 loss, resulting in apoptosis rather than senescence. FDX2 loss also sensitized cells to ferroptosis, as evidenced by compromised redox homeostasis of membrane phospholipids. Our results suggest that p53 status and phospholipid homeostatic activity are critical determinants of diverse biological outcomes of Fe-S deficiency in cancer cells.

Iron-sulfur clusters (Fe-Ss) are inorganic complexes of iron and sulfide found in multiple forms (Fe_2_S_2_, Fe_4_S_4_, and Fe_3_S_4_) in life forms from bacteria to humans. In vertebrates, Fe-Ss are synthesized from Fe^2+^ and cysteine substrates primarily in mitochondria by a core Fe-S assembly complex containing seven protein subunits, namely, NFS1, FDX2, ISCU2, FXN, ACP1, PLP, and ISD11 (1, 2, 3). Other proteins, such as FDXR, GLRX5, and ISCA1/2, are required for synthesis, transport and/or transfer of Fe-Ss to specific proteins (1, 2). Aberrant Fe-S biosynthesis is implicated in many diseases such as Friedreich’s ataxia and several types of myopathy (1, 2). Mutations in genes encoding the proteins listed above are seen in patients and thought to impair Fe-S biosynthesis and underlie a wide variety of phenotypes, including metabolic, neurological, or hematological disorders with multiorgan involvement. Moreover, recent studies reveal that cancer cells show high dependency on some Fe-S assembly factors in some contexts (4, 5, 6), implying that factors associated with Fe-S synthesis or its regulation could serve as a potential cancer targets.

A subgroup of proteins known as called Fe-S proteins requires Fe-Ss as cofactors for proper function and/or regulation (1, 2). These proteins bind Fe-Ss at specific Cys or His residues and play important roles in many cellular functions. Continuous and adequate Fe-S biosynthesis is essential for proper function of Fe-S proteins, as Fe-Ss are unstable in aqueous solution, difficult to store intracellularly, and remain labile even in protein/peptide-bound states due to oxidative stress. To date, ∼50 Fe-S proteins have been identified in humans, and their functions cover a broad range of cellular activities including respiration, DNA replication/repair, tRNA modification, nucleotide synthesis, heme synthesis, and iron regulation (1, 2, 7, 8, 9, 10, 11, 12, 13, 14, 15).

In the context of cancer, overexpression of NFS1, a component of the core Fe-S assembly complex, due to gene amplification is reported in lung cancer (4). Such NFS1 overexpression is suggested to be particularly important for adaptation to high oxygen conditions in the lung. NFS1 suppression *via* RNAi in a lung cancer line increases cellular-free Fe^2+^, predisposing cells to a form of Fe-dependent cell death called ferroptosis (4). However, it is unclear whether comparable phenotypes are common to Fe-S deficiency or whether decreased proliferation caused by NFS1 suppression is due solely to ferroptosis. On the other hand, recent studies report that loss/suppression of FDX2, another component of the core Fe-S assembly complex, by RNAi or genome-editing suppressed proliferation of some human cancer or immortalized lines (16, 17, 18). For example, Joshi *et al.* reported that *FDX2* KO decreases levels of many Fe-S proteins, including those functioning in the electron-transport chain (ETC) in HepG2 hepatoma cells, and that *FDX2*-KO HepG2 cells are negatively selected in cell culture conditions (17). However, how FDX2 loss alters cellular functions, including proliferation/survival, remains unclear in other forms of cancer.

Here, comparable to *NFS1* amplification in lung cancer, we observed *FDX2* gene amplification in a substantial fraction of ovarian cancer (OVC) cases, an observation not previously reported in other cancer types. Accordingly, we chose to analyze FDX2 activity in the context of OVC and developed a conditional FDX2 KO human OVC line, enabling us to analyze effects of FDX2 deficiency. Using this model system, we report biochemical and biological outcomes of FDX2 deficiency and define factors that modulate these phenotypes.

## Results

### FDX2 loss promotes senescence-like growth arrest of OVC cells

Analysis of The Cancer Genome Atlas (TCGA) dataset revealed that the frequency of *FDX2* alteration was highest in OVC compared to other cancer types (Fig. S1*A*), with amplification the most highly reported alteration. Dependency Map (DepMap) dataset analysis suggested that OVC cells showed the greater susceptibility to *FDX2*-KO than other pan-cancer groups (Fig. S1*B*). These results suggest that *FDX2*-KO decreases proliferation of all OVC cell lines in the dataset, and that the magnitude of the *FDX2*-KO effect is comparable among OVC histological subtypes (Fig. S1*C*).

**Figure 1:**
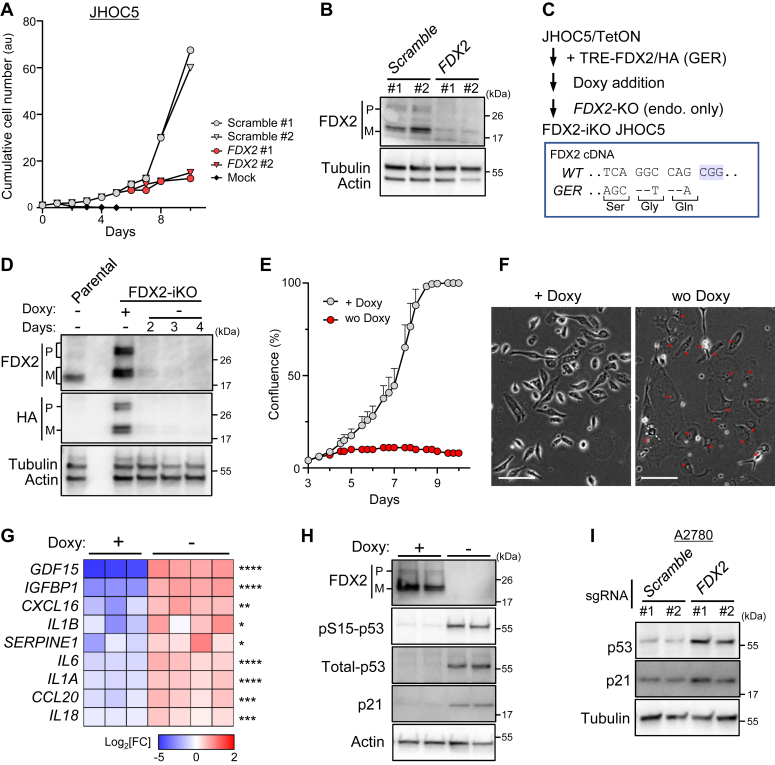
**FDX2 loss induces human OVC cell senescence.***A*, proliferation of JHOC5 cells transduced with Cas9 plus either FDX2 sgRNAs or control sgRNAs or mock-infected (mock). Shown are representative results of experiments repeated three times. *B*, Western blotting of cells indicated in *A*. In the FDX2 blot, P and M denote premature and mature forms of FDX2, respectively. *C*, schematic showing generation of FDX2-iKO cells. JHOC5 cells were first engineered to express exogenous HA-tagged (at the C terminus) FDX2 (FDX2/HA) once Doxy is added to cell cultures. Then, in the presence of Doxy the endogenous *FDX2* gene was knocked out by genome editing, such that Doxy withdrawal promoted loss of FDX2 protein. After establishment, cells were maintained in Doxy-containing medium. A portion of the FDX2 nucleotide sequence (GER, for genome-editing resistant) shown in the *box* (*bottom*) below harbors silent mutations within the FDX2 sgRNA target site. PAM sequence is highlighted in *blue*. *D*, Western blot of FDX2-iKO JHOC5 cells before and after Doxy withdrawal. P and M denote premature and mature forms of FDX2, respectively. The parental JHOC5 cell lysate is shown for comparison. *E*, proliferation of FDX2-iKO JHOC5 cells cultured with or without Doxy. Shown are representative results of three repeated experiments. *F*, representative phase contrast images of FDX2-iKO JHOC5 cells cultured 7 days with or without Doxy. *Asterisks* denote positions of cell nuclei. Scale bars represent 100 μm. *G*, expression of mRNA encoding indicated SASP factors in FDX2-iKO JHOC5 cells. Cells were cultured 5 days in the presence or absence of Doxy before RNA-seq analysis. n = 3 or 4 biological replicates. *H*, upregulation of either Ser15-phosphorylated (pS15) or total p53 and p21 protein levels in FDX2-iKO JHOC5 cells 6 days after FDX2-KO induction. Full results relevant to parental JHOC5 cells are provided as Fig. S2*F*. *I*, Western blot of A2780 cells transduced with Cas9 plus either *FDX2* sgRNAs or control sgRNAs. Data are presented as mean + SD (*E*). ∗*p* < 0.05, ∗∗*p* < 0.01, ∗∗∗*p* < 0.001, and ∗∗∗∗*p* < 0.0001 as determined by two-tailed *t* test (*G*). Source data and exact *p* values are provided as a Source Data file. Doxy, doxycycline; HA, hemagglutinin; iKO, inducible KO; OVC, ovarian cancer; SASP, senescence-associated secretory phenotype; sgRNA, single guide RNA.

Next, to knockout *FDX2* in OVC cells, we transduced the human JHOC5 OVC line with lentivirus-expressing Cas9 and *FDX2* single guide RNA (sgRNA) and assessed changes in proliferation based on cell number. The JHOC5 line exhibits functional p53 protein and is widely used in the field (19, 20). Both *FDX2* transcript levels (Fig. S1*D*) and *FDX2* dependency of the JHOC5 line (Fig. S1*E*) were close to the average among OVC lines in the CCLE collection (Fig. S1, *D* and *E*). Cells transduced with *FDX2* sgRNA showed decreased proliferation relative to scramble controls (Fig. 1*A*), confirming that FDX2 is essential for maximum proliferation of this line, although KO efficiencies were partial in every analysis (Fig. 1*B*). Note that in these studies we observed two forms of FDX2 protein (denoted by “P” and “M” in Fig. 1*B*), as previously reported (17), likely due to posttranslational cleavage of its N-terminal mitochondrial targeting peptide.

To further assess FDX2 function, we established FDX2 conditional-KO JHOC5 cells by deleting the endogenous gene and overexpressing a tagged, inducible form of FDX2 protein. To do so, we overexpressed hemagglutinin (HA)-tagged FDX2 in JHOC5 cells in a doxycycline (Doxy)-dependent manner and then, in the presence of Doxy, selectively inactivated the endogenous gene by genome editing. (Fig. 1*C*, see Experimental procedures for details). Western analysis indicated robust expression of HA-tagged FDX2 protein in cells cultured with Doxy (Fig. 1*D*, lane 3). However, 4 days after Doxy withdrawal, FDX2 protein was undetectable by Western analysis (Fig. 1*D*). Conversely, within a day of Doxy restoration, FDX2 expression was rapidly restored (Fig. S2*A*). We then assessed proliferation of FDX2-inducible KO (iKO) cells cultured 10 days in the absence of Doxy and observed significant growth arrest relative to Doxy-treated control cells (Figs. 1*E* and S2*B*), indicating that FDX2 is essential for JHOC5 cell proliferation.

Morphologically, FDX2-deficient JHOC5 cells appeared enlarged and flattened but rarely showed signs of cell death, such as membrane rupture or lysis (Figs. 1*F* and S2*C*), suggesting that cells were undergoing senescence. To assess this possibility in FDX2-iKO JHOC5 cells, we initially evaluated activity of SA-β-gal, a marker of senescence, in cells cultured 7 days without Doxy but did not detect SA-β-gal activity (data not shown). However, transcripts encoding senescence-associated secretory phenotype (SASP) factors (21, 22, 23), such as interleukin (IL)-6, IL-1A/B, and IL-18, were upregulated in cells cultured 5 days without Doxy (Fig. 1*G*), and in comet assays we observed increased evidence of DNA damage in cells depleted of FDX2 by Doxy withdrawal relative to control cells (Fig. S2, *D* and *E*). Furthermore, FDX2-depleted cells showed p53 and p21 upregulation relative to control cells (Figs. 1*H* and S2*F*). Collectively, these results suggest that FDX2 depletion activates the DNA damage response (DDR) and p53/p21 signaling in JHOC5 cells, resulting in senescence-like growth arrest.

We also asked whether FDX2 loss induces p53 activation in OVC lines other than JHOC5. To do so, we transduced A2780 cells, a human p53-proficient OVC line, with Cas9/FDX2 sgRNAs and observed slowed proliferation coinciding with p53 upregulation (Figs. 1*I*, and S2, *G* and *H*), suggesting that p53 activation by FDX2 loss is a common phenotype in p53-proficient cells. Others previously reported that either heterozygous *FDX2* KO or siRNA-mediated FDX2 knockdown lowered basal p53 levels in HCT-116 cells (24). HCT116 cells show relatively low FDX2-dependence compared to other cell lines, including most OVC lines in the DepMap dataset (Fig. S2*I*). We found that neither FDX2 overexpression nor transient FDX2 suppression by siRNA altered p53 protein levels in OVC cells tested (Fig. S2, *J* and *K*). We conclude that the FDX2/p53 interplay previously seen in HCT-116 cells is likely context-dependent and does not occur in OVC lines tested and that p53 induction seen after FDX2 loss requires prolonged FDX2 inactivation.

### FDX2 loss promotes global downregulation of Fe-S proteins

**Figure 2:**
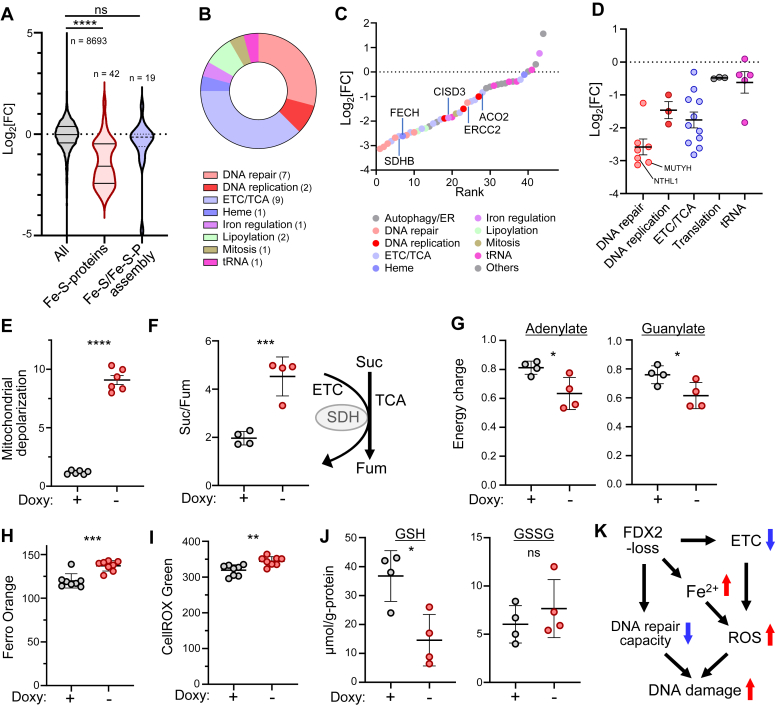
**FDX2 loss downregulates Fs-S protein and perturbs multiple cellular functions, likely converging on DNA damage.***A*, results of MS-based proteome analysis performed in FDX2-iKO cells cultured either with Doxy or after 5 or 6 days without Doxy to induce FDX2 loss. Shown are effects of FDX2 loss on all detected proteins, on all Fe-S proteins, or on proteins functioning in either Fe-S assembly or Fe-S protein assembly. *B*, classification and proportion of Fe-S proteins showing significantly changed levels (1 < |Log_2_[FC]|, *p* < 0.05) after FDX2 loss. Note that no Fe-S proteins show significant upregulation. *C*, rank plot of all Fe-S proteins detected in proteome analysis. Fe-S proteins were ranked based on degree of change in their levels after FDX2 depletion. Each Fe-S protein is color-coded to indicate its function. *D*, comparison of effects of FDX2 loss on Fe-S protein levels in indicated functional categories. *E*, analysis of mitochondrial depolarization using a JC-10 probe in FDX2-iKO JHOC5 cells in the presence or absence of Doxy. n = 6 biological replicates. *F*, succinate/fumarate ratio of indicated FDX2-iKO cells cultured 5 days in the presence or absence of Doxy (*left*). Schematic illustrates succinate conversion to fumarate in the TCA cycle catalyzed by the succinate dehydrogenase (SDH) complex, an activity coupled to that of the ETC complex II (*right*). n = 4 biological replicates. *G*, adenylate (*left*) and guanylate (*right*) energy charges of FDX2-iKO cells grown in the presence or absence of Doxy. n = 4 biological replicates. *H*, cellular-free Fe^2+^ levels of FDX2-iKO cells cultured 5 days in the presence or absence of Doxy. n = 8 biological replicates. *I*, cellular ROS levels of FDX2-iKO cells cultured 5 days in the presence or absence of Doxy. n = 8 biological replicates. *J*, levels of GSH (*left*) and GSSG (*right*) in FDX2-iKO cells cultured 5 days in the presence or absence of Doxy. n = 4 biological replicates. *K*, proposed model of FDX2 deficiency–induced DNA damage. See the text for details. Data are presented as mean + SEM (*A*, *D*, and *E–J*). ∗*p* < 0.05, ∗∗*p* < 0.01, ∗∗∗*p* < 0.001, and ∗∗∗∗*p* < 0.0001 as determined by one-way ANOVA with Tukey’s post hoc test (*A*) or two-tailed *t* test (*E–J*). Source data and exact *p* values are provided as a Source Data file. Doxy, doxycycline; ETC, electron-transport chain; Heme, heme biosynthesis; iKO, inducible KO; MS, mass spectrometry; ns, not significant; ROS, reactive oxygen species; TCA, tricarboxylic acid cycle; tRNA, tRNA synthesis and modification.

Others have reported that suppression of core Fe-S assembly complex subunits, such as NFS1 and FDX2, decreases levels of several Fe-S proteins, including ETC components (16, 17, 25). To evaluate global effects of FDX2 loss on cellular Fe-S proteins, we conducted mass spectrometry–based proteome analysis of FDX2-iKO JHOC5 cells, before and after 4 or 5 days of Doxy withdrawal to induce FDX2 deficiency. FDX2 was one of the most markedly reduced proteins in cells cultured without Doxy (Fig. S3*A*). Proteome analysis in both conditions identified 8695 other proteins, among which 614 and 278 were significantly downregulated and upregulated (Log_2_[FC] > 1, *p* < 0.05), respectively, following Doxy withdrawal and subsequent FDX2 deficiency (Fig. S3*A*). Among global proteomic changes seen following FDX2 loss, effects on Fe-S proteins were particularly robust (Figs. 2*A* and S3*A*). Specifically, among the 42 Fe-S proteins detected in both the presence or absence of Doxy, 25 (including FDX1) were significantly downregulated by Doxy withdrawal (Fig. S3*B* and Table S1).

Several functional categories of Fe-S proteins were significantly downregulated by FDX2 deficiency, with DNA repair and ETC/tricarboxylic acid cycle proteins predominant (Fig. 2*B*). The extent of down-regulation differed among Fe-S proteins but was most evident in Fe-S proteins associated with DNA repair (Fig. 2, *C* and *D*), findings confirmed by Western blot analysis (Fig. S3*B*). Fe-S proteins downregulated in this category included MUTYH and NTHL (Fig. 2*D*), both essential for base-excision repair, an activity essential for repair of reactive oxygen species (ROS)-induced DNA damage (26). Interestingly, despite the significant decrease in levels of many Fe-S proteins after FDX2 depletion, their mRNA levels overall remained unchanged (Fig. S3*C*), suggesting that posttranscriptional mechanism(s) account for Fe-S protein downregulation. By contrast, levels of Fe-S and/or Fe-S protein assembly factor proteins were almost unchanged by FDX2 depletion, with some exceptions: ISCA1 and GLRX5 protein levels decreased following FDX2 depletion, while FDXR protein levels were significantly upregulated (Fig. S3*D* and Table S2).

### Cellular functions perturbed by FDX2 loss converge on DNA damage

Given the findings reported above, we hypothesized that down-regulation of multiple ETC Fe-S proteins in FDX2-deficient conditions might disrupt mitochondrial respiration capacity. Accordingly, we observed depolarization of the mitochondrial membrane (Fig. 2*E*) and an increase in the succinate/fumarate ratio (Figs. 2*F* and S3*E*) in FDX2-iKO JHOC5 cells cultured without Doxy, indicative of perturbed mitochondrial ETC/TCA function. In agreement, the cellular energy charge also decreased after FDX2 loss (Fig. 2*G*).

We then asked whether FDX2 loss alters intracellular Fe^2+^ levels, given that FDX2 loss decreased levels of FECH and CISD3 proteins, which function in heme synthesis and iron regulation, respectively (Fig. 2, *C* and *D*). Accordingly, we detected higher levels of both cellular and mitochondrial-free Fe^2+^ in FDX2-depleted relative to control cells, confirming an impact on iron homeostasis (Figs. 2*H* and S3*F*).

ETC dysfunction reportedly coincides with increased levels of ROS in many cell types (27). Also, cellular-free Fe^2+^ reacts with H_2_O_2_ (the Fenton reaction) to generate hydroxyl radicals to promote ROS production (28, 29) (Fig. S3*G*). Strikingly, we observed significantly higher levels of cellular or mitochondrial ROS in JHOC5 cells depleted of FDX2 than control cells (Figs. 2*I* and S3*H*). Consistently, GSH levels markedly decreased in FDX2-depleted relative to control cells, while GSSG levels were similar (Fig. 2*J*). Overall, these results strongly suggest that FDX2 loss promotes increased ROS production and impairs DNA repair capacity, both of which converge on DNA damage (Fig. 2*K*).

### p53 status governs the fate of FDX2-deficient OVC cells

*TP53* encodes the tumor suppressor p53 and is the most frequently mutated gene in OVC (30). Our results reported above suggest that p53 functions in growth arrest of FDX2-iKO JHOC5 cells. Paradoxically, DepMap dataset analyses showed a negative impact of FDX2 deficiency on cell proliferation, even in p53-deficient lines (Fig. S1, *B* and *C*). We confirmed that *FDX2* KO suppressed proliferation of the OVC line ES2, which harbors a loss-of-function *TP53* mutation (S241F) (Fig. S4, *A* and *B*), suggesting that FDX2 loss also suppresses OVC cell growth p53 independently.

**Figure 3:**
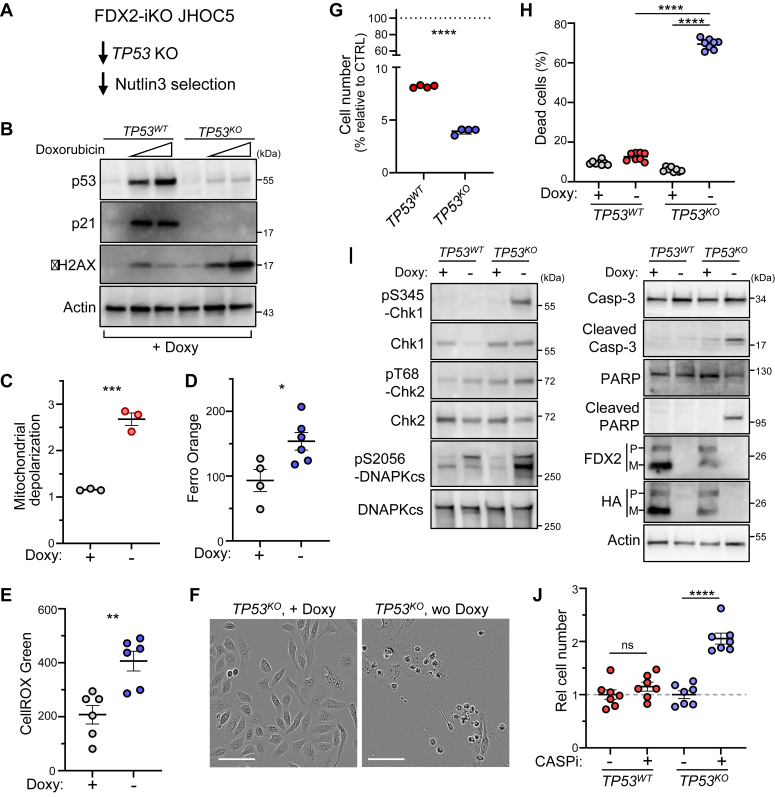
**p53 deficiency boosts DNA damage responses elicited by FDX2 loss, leading to apoptotic cell death.***A*, establishment of *TP53*^*KO*^ FDX2-iKO JHOC5 cells. *B*, Western blot analysis of indicated proteins in parental (*TP53*^*WT*^) and *TP53*^*KO*^ FDX2-iKO cells maintained in Doxy and treated 20 h with doxorubicin at 0, 0.1, or 0.3 μM. *C*, analysis of mitochondrial depolarization using a JC-10 probe in *TP53*^*KO*^ FDX2-iKO JHOC5 cells cultured 5 days in the presence or absence of Doxy. n = 3 biological replicates. *D*, cellular-free Fe^2+^ levels in *TP53*^*KO*^ FDX2-iKO cells cultured 5 days in the presence or absence of Doxy. n = 8 biological replicates. *E*, cellular ROS levels in *TP53*^*KO*^ FDX2-iKO cells cultured 5 days in the presence or absence of Doxy. n = 8 biological replicates. *F*, phase-contrast image of *TP53*^*KO*^ FDX2-iKO cells cultured 8 days with or without Doxy, the latter to induce FDX2 loss. Scale bars represent 100 μm. *G*, comparison of numbers of viable cells in *TP53*^*WT*^ and *TP53*^*KO*^ FDX2-iKO lines after 8 days cultivation in the absence of Doxy. CTRL, cells cultured in the presence of Doxy. n = 4 biological replicates. *H*, viability of cells cultured 8 days in the presence or absence of Doxy. n = 8 biological replicates. *I*, activation status of DDR and caspase-3 based on Western blotting of *TP53*^*WT*^ and *TP53*^*KO*^ FDX2-iKO cells cultured 7 days in the presence or absence of Doxy. Actin serves as loading control. *J*, effects of caspase inhibition (CASPi) on numbers of viable *TP53*^*WT*^ and *TP53*^*KO*^ FDX2-iKO cells cultured 8 days in the presence or absence of doxycycline. Cells were treated with the pan-caspase inhibitor Z-VAD-FMK or control vehicle starting at day 5 of analysis. n = 7 biological replicates. Data are presented as mean + SEM (*C–E*, *G*, *H*, and *J*). ∗*p* < 0.05, ∗∗*p* < 0.01, and ∗∗∗∗*p* < 0.0001 as determined by two-tailed *t* test (*C–E*, *G*, and *J*) or one-way ANOVA with Tukey’s post hoc test (*H*). Source data and the exact *p* values are provided as a Source Data file. Doxy, doxycycline; iKO, inducible KO; ns, not significant; ROS, reactive oxygen species.

To assess how p53 deficiency modulates phenotypes seen after FDX2 loss, we used genome editing to knockout *TP53* in FDX2-iKO JHOC5 cells and then negatively selected p53-proficient cells using Nutlin-3, a p53 activator (31) (Fig. 3*A*). That analysis confirmed that resulting cells had lost p53 function, based on Nutlin-3 insensitivity (Fig. S4*C*). Unlike p53-proficient parental cells (*TP53*^*WT*^ FDX2-iKO JHOC5), *TP53*^*KO*^ cells did not show p21 upregulation when treated with doxorubicin to induce DNA damage, a finding confirmed by increased γH2AX levels (Fig. 3*B*). After Doxy was removed from the medium, *TP53*^*KO*^ FDX2-iKO JHOC5 cells showed FDX2 loss (Fig. S4*D*) and Fe-S protein downregulation (Fig. S4*E*), decreased proliferation (Fig. S4*F*), depolarization of mitochondria (Fig. 3*C*), and increased levels of free Fe^2+^ and ROS (Fig. 3, *D* and *E*), as did *TP53*^*WT*^ FDX2-iKO JHOC5 cells. We also observed SASP activation in *TP53*^*KO*^ FDX2-iKO JHOC5 cells after Doxy-withdrawal (Fig. S4*G*), findings consistent with the idea that SASP is generally p53-independent (32). Overall, most phenotypes promoted by FDX2 deficiency seen in a p53-proficient context (summarized in Fig. 2*K*) are also exhibited by *TP53*^*KO*^ FDX2-iKO cells. However, FDX2 loss promotes p53 induction in *TP53*^*WT*^ but not *TP53*^*KO*^ cells (Fig. S4*H*).

Importantly, when we removed Doxy to induce FDX2 loss in *TP53*^*KO*^ FDX2-iKO JHOC5 cells, a large fraction of cells exhibited morphologies seen in dying cells (Fig. 3*F*), and the number of viable cells significantly decreased in those *TP53*^*KO*^ cells relative to cells parental cells (Fig. 3, *G* and *H*). These findings suggest that after p53 loss, phenotypes associated with FDX2 loss in JHOC5 cells shift from senescence to cell death.

To assess this possibility, we analyzed mechanisms underlying FDX2 deficiency–induced cell death in *TP53*^*KO*^ FDX2-iKO JHOC5 cells. Given that p53 loss of function reportedly promotes relatively severe DNA damage (33), we evaluated activation status of proteins functioning in the DDR in both *TP53*^*WT*^ and *TP53*^*KO*^ FDX2-iKO JHOC5 cells. After inducing FDX2 loss, we observed higher levels of phosphorylated CHK1, CHK2, and DNA-PKcs in *TP53*^*KO*^ FDX2-iKO relative to *TP53*^*WT*^ FDX2-iKO cells (Fig. 3*I*). Unexpectedly, CHK1 protein levels decreased after FDX2 loss in *TP53*^*WT*^ but not *TP53*^*KO*^ FDX2-iKO JHOC5 cells. Moreover, we observed caspase-3 activation, based on its cleavage and generation of cleaved forms of PARP, a caspase substrate, only in *TP53*^*KO*^ FDX2-depleted JHOC5 cells (Fig. 3*I*). *TP53*^*KO*^ FDX2-iKO cells showed increased annexin V binding activity after Doxy withdrawal, whereas parental JHOC5 and *TP53*^*WT*^ FDX2-iKO cells did not (Fig. S4*I*). *TP53*^*KO*^ FDX2-iKO cells also exhibited DNA fragmentation after FDX2 loss, based on TdT-mediated dUTP nick-end labeling (TUNEL) analysis (Fig. S4*J*). Overall, these findings indicate that *TP53*^*KO*^ FDX2-iKO cells exhibit apoptotic phenotypes upon FDX2 depletion. Furthermore, treatment of cells with the pan-caspase inhibitor Z-VAD-FMK increased the number of viable FDX2-deficient cells cultured without Doxy on a *TP53*^*KO*^ but not a *TP53*^*WT*^ background (Fig. 3*J*). Collectively, these results suggest that DNA damage following FDX2 loss is enhanced in p53-deficient conditions, which, in turn, promotes apoptotic cell death *via* caspase activation.

### FDX2 loss predisposes cells to ferroptosis in both p53-proficient and p53-deficient conditions

Based on findings reported above, we posit that FDX2 deficiency promotes senescence in p53-proficient JHOC5 cells but apoptosis in the context of p53 deficiency. Given that impaired Fe-S biosynthesis, however, reportedly induces ferroptosis in some contexts (4, 5, 28), we also asked whether FDX2 loss affects susceptibility to ferroptosis.

**Figure 4:**
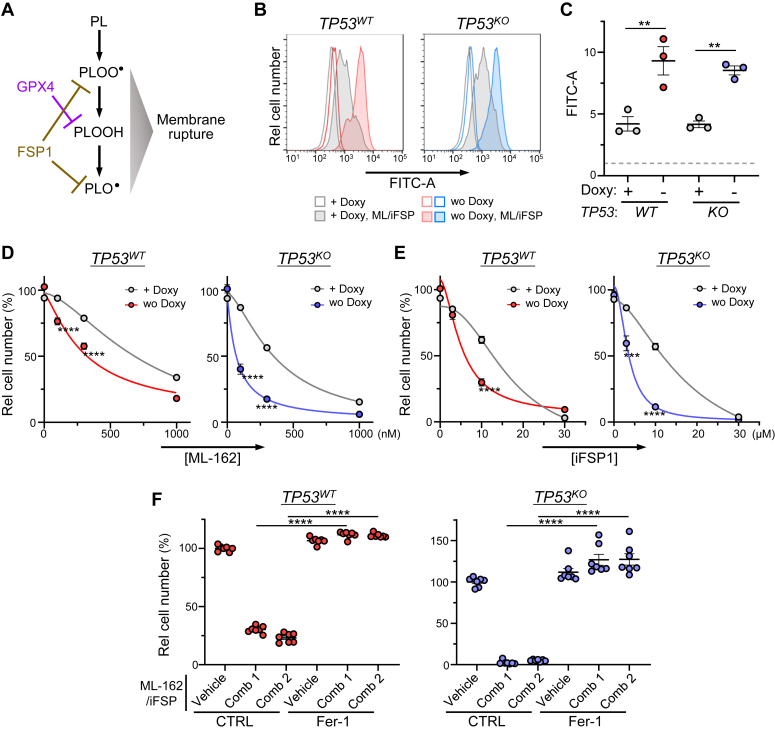
**FDX2 loss predisposes cells to ferroptosis in both p53-proficient and p53-deficient conditions.***A*, scheme showing phospholipid (PL) oxidation associated with rupture of the cell membrane leading to ferroptosis. GPX4 and FSP1 protect cells from ferroptosis by decreasing PLOOH levels and scavenging PL radicals, respectively. *B*, analysis of lipid peroxidation in FDX2-iKO JHOC5 cells. Cells were cultured 4 days with or without Doxy, treated with ferroptosis-inducing reagents (ML-162 and iFSP1 at 0.3 and 3 μM, respectively) or left untreated for 2 h, and then incubated with BODIPY C11 probe. Fluorescent signals of oxidized BODIPY C11 were detected by flow cytometry. Shown is one representative of three independent experiments. Note that *TP53*^*WT*^ and *TP53*^*KO*^ cells were analyzed simultaneously. *C*, statistical analysis of the three independent experiments reported in B. Fluorescent intensities of each sample (mean value) are shown as a value relative. Values seen in *TP53*^*WT*^ cells cultured in the presence of Doxy (not stimulated with GPX4/FSP1 inhibitors) were arbitrarily defined as 1.0 (*dashed line*). *D*, effects of FDX2 deficiency on GPX4 inhibition of *TP53*^*WT*^ and *TP53*^*KO*^ FDX2-iKO cells. Cells were cultured with or without Doxy for 4 days and treated with the maximum dose of an FSP1 inhibitor (30 μM iFSP1) plus a GPX-4 inhibitor (ML-162) at indicated concentrations for an additional day. The number of viable cells was determined by sulforhodamine B staining. n = 7 to 8 biological replicates for each group. *E*, effects of FDX2 deficiency on FSP1 inhibition of *TP53*^*WT*^ and *TP53*^*KO*^ FDX2-iKO cells. Cells were cultured 4 days with or without Doxy, treated with the maximum dose of ML-162 (1 μM) plus iFSP1 at indicated concentrations for an additional day, and analyzed as in *D*. n = 7 to 8 biological replicates for each group. *F*, effects of ferrostatin-1 (Fer-1) on death of FDX2-iKO cells induced by dual inhibition of GPX4 and FSP1. Fer-1 was used at 1 μM. Data are presented as mean + SEM (*C–F*). ∗∗*p* < 0.01, ∗∗∗*p* < 0.001, and ∗∗∗∗*p* < 0.0001 as determined by two-tailed *t* test (*D* and *E*) or one-way ANOVA with Tukey’s post hoc test (*C* and *F*). Source data and the exact *p* values are provided as a Source Data file. Doxy, doxycycline; iFSP, inhibitor of FSP; iKO, inducible KO.

Peroxidized phospholipids (PLs) and PL radicals reportedly lead to cell membrane rupture and cell death (34, 35), and GPX4 and FSP1 participate in critical antiferroptotic pathways that protect PLs from Fe^2+^-dependent peroxidation (Fig. 4*A*). Thus, we treated FDX2-iKO JHOC5 cells cultured in the presence of Doxy either with a GPX4 inhibitor (ML-162) or with an inhibitor of FSP1 (iFSP); however, we did not observe significant cell death (Fig. S5*A*). However, when we treated cells with a combination of ML-162 and iFSP (also in the presence of Doxy), FDX2-iKO JHOC5 cells underwent cell death (Fig. S5*A*), consistent with the likely redundant roles played by GPX4 and FSP1 in suppressing ferroptosis. In comparable analysis, we also examined PL peroxidation activity in FDX2-deficient cells using BODIPY C11 assays. We observed that in absence of ML-162 and iFSP1 treatment, both FDX2-expressing (Doxy-treated) and FDX2-deficient (cultured 4 days without Doxy) cells showed comparable levels of oxidized BODIPY, irrespective of *TP53* status (Fig. S5*B*). However, in the presence of Doxy, 2 h of culture with both ML-162 (0.3 μM) and iFSP1 (3 μM) increased levels of oxidized BODIPY relative to untreated cells, and those levels increased even further after Doxy removal to block FDX2 expression, both in p53-deficient and p53-proficient conditions (Fig. 4, *B* and *C*). Note that treatment of *TP53*^*WT*^ or *TP53*^*KO*^ FDX2-iKO JHOC5 cells with both ML-162 (0.3 μM) and iFSP1 (3 μM) kills ∼ 20% or ∼40% of cells, respectively (Fig. S5*A*). These results indicate that FDX2 deficiency renders cells susceptible to PL peroxidization, an outcome that becomes apparent when GPX4 and FSP1 function is impaired.

We next cultured FDX2-iKO cells 4 days with or without Doxy, treated both groups for 1 day with either ML162 or iFSP1 at various concentrations, and then counted viable cells in each group. Those analyses indicated that inhibition of GPX4 or FSP1 alone was not sufficient to induce cell death even after FDX2 loss (Fig. 4, *D* and *E*). However, analysis of cells treated with combined ML162 and iFSP1 indicated that FDX2 loss promoted cell death, even at relatively low inhibitor concentrations (Fig. 4, *D* and *E*). At the maximum dose (1 μM ML-162 plus 30 μM iFSP1), cell death promoted by FDX2 loss (see differences between “+ Doxy” and “wo Doxy” (Fig. 4, *D* and *E*)) were minor or rarely observed. Interestingly, death of FDX2-deficient JHOC5 cells treated with ML162 plus iFSP1 was blocked by cotreatment with the ferroptosis inhibitor ferrostatin-1 (Fer-1) (Fig. 4*F*), suggesting that FDX2 loss sensitizes JHOC5 cells to ferroptosis, an activity revealed only when antiferroptosis activities become compromised.

## Discussion

Here, we report biological outcomes following loss of FDX2, a component of the core Fe-S assembly factor, as well as other factors that influence OVC cells. We confirmed that FDX2 loss induces widespread downregulation of Fe-S proteins, as reported previously (17). We further revealed consequences of Fe-S protein downregulation, namely deficiencies in respiration, iron regulation and redox homeostasis. Our results suggest that many of these abnormalities converge on DNA damage, which, in turn, drives either senescence or apoptosis programs. We also found that FDX2 loss predisposes cells to ferroptosis, although this phenotype became apparent only when cellular antiferroptotic activities were impaired (Fig. S6).

Overall, we propose that FDX2-iKO cells could be a unique tool to study Fe-S assembly, since these cells can be easily shifted to an FDX2-deficient state and back to a normal state by respective removal and addition of Doxy. These properties allowed us to overcome cell viability challenges often encountered in loss-of-function analysis of essential genes and to evaluate consequences of FDX2-depletion in more detail than previously reported after siRNA-mediated knockdown or constitutive gene KO analyses (16, 17, 18, 24). Fe-S binding to a protein often stabilizes that protein’s structure (1, 2, 9, 15, 36). Conversely, FDX2 loss promoted global downregulation of Fe-S proteins primarily at posttranscriptional levels (Fig. 2, *A*–*D*). That downregulation may be triggered by oxidation and subsequent disintegration of Fe-S bound to such proteins. However, the extent of downregulation was not uniform among Fe-S proteins: after FDX2 loss, many Fe-S proteins associated with DNA repair showed significant downregulation, while those functioning in translation and tRNA modification showed little change in protein levels (Fig. 2*D*). Currently, the reasons for these differences remain unclear. However, this study revealed several biochemical consequences after global downregulation of Fe-S proteins in proliferating cells. FDX2 loss, likely through Fe-S deficiency, promoted ETC dysfunction (Fig. 2, *E*–*G*) and high cellular Fe^2+^ levels (Fig. 2*H*), both of which boost ROS production (Fig. 2, *I* and *J*) and promote DNA damage (Figs. 3*I* and S2, *D* and *E*). Downregulation of the Fe-S proteins MUTYH and NTHL (Fig. 2*D*), both essential for base-excision repair, following FDX2 depletion must prevent efficient repair of ROS-induced DNA lesions. Collectively, we propose that perturbation of numerous cellular functions following FDX2 deficiency converge on DNA damage (Fig. 2*K*).

In our FDX2-iKO model, p53 status determines cell fate following FDX2 deficiency. Cellular senescence induced by FDX2 loss depends on functional p53, as is seen in other models of senescence (37). Without p53, we observed an enhanced DDR and caspase activation (Fig. 3*I*), likely because DNA damage is more severe in p53-deficient *versus* p53-proficient cells, and our findings are consistent with the fact that the p53/p21 pathway antagonizes DNA damage and caspase signaling and has a cellular prosurvival function under stressed conditions (33). Relevant to DDR in FDX2-depleted cells, downregulation of CHK1 protein itself in *TP53*^*WT*^ cells but not *TP53*^*KO*^ cells was unanticipated (Fig. 3*I*), and mechanisms underlying this outcome should be evaluated in future studies. We also note that the consequences of p53 activation depend on context (38). For example, in JHOC5 cells, p53 activation by Nutlin-3 led to senescence, as seen in cells undergoing FDX2 loss, but Nutlin-3 treatment induced massive cell death in p53-proficient A2780 OVC cells (data not shown). Thus, we do not exclude the possibility that FDX2 loss can promote cell death, even in p53-proficient cancer cells.

FDX2 loss in JHOC5 cells led to senescence or apoptosis, depending on p53 status. However, FDX2 loss in JHOC5 cells did not promote ferroptosis, unless cells were subjected to dual GPX4/FSP1 inhibition (Fig. 4). These results suggest that FDX2 loss predisposes cells to ferroptosis as previously reported after NFS1 loss in the A549 lung cancer line (4). Our results indicate that JHOC5 cells exhibit sufficient antiferroptotic activity to overcome Fe^2+^ and/or ROS overload induced by FDX2 loss. Interestingly, many recent studies suggest that high resistance to ferroptosis is a hallmark of cancer (39, 40, 41). Collectively, the consequences of Fe-S biosynthesis deficiency in many cancer cells would be primarily senescence or apoptosis rather than ferroptosis. Nonetheless, we anticipate that inhibition of FDX2 function/expression may cause ferroptosis if antiferroptotic activities are low due to cell-intrinsic or non–cell-intrinsic reasons (42, 43). Importantly, we observed a predisposition to ferroptosis following FDX2 loss in p53-proficient and p53-deficient conditions (Fig. 4). We also note that either hypoxia or hyperoxia may modulate the impact of FDX2 deficiency given that Fe-Ss are oxygen-labile (9, 44). Effects of FDX2 loss on hypoxia remain to be addressed.

Gene amplification revealed by our analysis of TCGA cohorts suggests a possible cancer-promoting role for FDX2 in OVC (Fig. S1*A*), analogous to NFS1 in lung cancers. Indeed, we found that FDX2 is indispensable for OVC cell proliferation, suggesting that FDX2 could serve as a potential therapeutic target, particularly in OVC. In these contexts, knowing how large the therapeutic window is between cancer and noncancer cells/tissue is essential. Mutation of the gene encoding FXN, a subunit of core Fe-S assembly complex, causes Friedreich’s ataxia. In addition, mutation(s) or other abnormalities in genes encoding Fe-S proteins or assembly factors have been implicated in various diseases (1, 2, 44). Therefore, targeting Fe-S assembly to treat cancer remains challenging. However, accumulating evidence suggests that the extent of deficiency in Fe-S assembly factors varies in normal cells (1, 2). Relevant to FDX2, one myopathy patient reportedly harbored a homozygous c1A > T mutation in *FDX2*, which disrupts the ATG translation initiation codon and severely decreases FDX2 protein levels, but that patient recovered following treatment of symptoms (45). Nevertheless, further study is needed to evaluate the possibility of targeting FDX2 therapeutically in cancer.

In summary, our results show that the biochemical and biological outcomes of FDX2 deficiency in the context of OVC depend on p53 and antiferroptotic activities. Further studies are needed to understand how FDX2 deficiency affects healthy normal cells/tissues in order to develop novel strategies to therapeutically target Fe-S and related metabolism in ovarian and other cancers.

## Experimental procedures

### Reagents

Fluorescent probes CellROX Green, MitoSOX Red, and BODIPY-C11 were purchased from Thermo Fisher Scientific. FerroOrange, Mito-FerroGreen, and JC-10 were purchased from Dojindo. ML-162 was obtained from Cayman. iFSP1 and Fer-1 was purchased from MedChemExpress.

### Cell culture

Human 293T cells were obtained from Riken Bioresource Center. The human OVC lines JHOC5, ES2, and A2780 were kindly provided by Carla Grandori (Fred Hutchinson Cancer Research Center) (20). All OVC lines were maintained in high-glucose Dulbecco's modified Eagle medium supplemented with 10% fetal calf serum. 293T cells were cultured in RPMI1640 with 10% fetal calf serum. All lines were verified as mycoplasma-free using a MycoAlert kit (Lonza). Cell line authentication test were not performed.

### Lentivirus-mediated gene expression

Lentivirus was produced in 293T cells using standard procedures with FuGENE HD (Promega), psPAX2 and pMD2.G packaging plasmids (CELLECTA), and lentivirus plasmids, as described (46). Lentivirus plasmids pLV-TRE-HA/FDX2 (GER, genome-editing resistant)-Neo and LV-tTS/rtTA-Hyg were sourced from Vector Builder. Unless specified, virus infections were performed using a 1:1 to 2 mixture of 293T culture supernatant and fresh medium. Polybrene was added to the virus mixture at 8 ug/ml to promote infection.

### Analysis of cancer DepMap and TCGA datasets

We used the dataset published in the release of “DepMap Public 22Q1” (https://depmap.org/portal/download/) to assess how KO of *FDX2* by genome-editing impacts proliferation of cell lines collected in the CCLE panel, as described (47, 48). We used the same dataset to analyze *FDX2* mRNA levels. Alteration frequency of the *FDX2* gene in clinical cancer samples was assessed over cohorts of “curated set of nonredundant studies” published in TCGA (https://www.cancer.gov/ccg/research/genome-sequencing/tcga). The dataset includes 214 manually curated studies, including TCGA and non-TCGA studies, with no overlapping samples. Results were summarized by cancer-type.

### *FDX2* KO by lentivirus-based CRISPR/sgRNA constructs

Lentivirus plasmids expressing both Cas9:Neo and *FDX2* sgRNAs and appropriate control constructs were purchased from VectorBuilder. Lentivirus production and infection were performed as described above. Infected cells were selected in 1000 μg/ml Geneticin.

### Cell number determinations

Cell proliferation was continuously monitored using the IncuCyte cell analyzer (Sartorius). Alternatively, relative cell numbers at endpoints were determined by sulforhodamine B staining, as described (46).

### FDX2 iKO cells

JHOC5 cells were transduced with tTS and rtTA using a lentivirus vector to produce JHOC5-TetOn cells. Cells were selected in 250 μg/ml hygromycin and then transduced with a gene cassette–expressing FDX2 (for GER)/HA cDNA driven by the TRE promotor using a lentivirus vector. FDX2 (GER) carries silent mutations at the FDX2_CC2 sgRNA target site. Resulting JHOC5-TetON-FDX2 (GER)/HA cells were selected in 1000 μg/ml Geneticin. To delete the endogenous gene, we transfected JHOC5-TetON-FDX2 (GER)/HA cells grown in the presence of 100 ng/ml Doxy with RNA-polypeptide (RNP) complexes consisting of Cas9 protein and FDX2_CC2 sgRNA using LipofectAMINE CRISPRMAX reagent (Thermo Fisher Scientific), based on the manufacturer’s instructions. sgRNA FDX2_CC2 was designed using the ChopChop program (https://chopchop.cbu.uib.no/) and synthesized by GenScript. The FDX2_CC2 sequence is as follows; 5′- mU∗mC∗mG∗rUrArGrArCrCrGrCrUrCrArGrGrCrCrArGrGrUrUrUrUrArGrArGrCrUrArGrArArArUrArGrCrArArGrUrUrArArArArUrArArGrGrCrUrArGrUrCrCrGrUrUrArUrCrArArCrUrUrGrArArArArArGrUrGrGrCrArCrCrGrArGrUrCrGrGrUrGrCrU∗mU∗mU∗mU-3′ (m and ∗ denote 2' O-methyl RNA and phosphorothioate, respectively). RNP-transfected cells were subjected to a standard limiting dilution cloning procedure in medium supplemented with 100 ng/ml Doxy, and KO clones were identified by Western blotting. Three FDX2-iKO clones were established, and their phenotypic similarities were confirmed. After establishment, clones were cultured in 30 to 100 ng/ml Doxy. Unless stated, results using clone #1 were shown in this paper.

To induce FDX2 deficiency, cells were precultured 3 to 4 days with or without Doxy and seeded into new dishes or plates. For FDX2 reexpression experiments, Doxy was added to the medium on day 5. A real-time cell proliferation assay was performed using IncuCyte.

### Western blot analysis and capillary-based immunoassays

Cells were lysed by sonication using a BIORUPTOR device (SonicBio Co) in radio-immunoprecipitation assay buffer supplemented with protease inhibitors. Lysate protein concentrations were determined using a detergent compatible assay kit (Bio-Rad). SDS-PAGE was performed using 4 to 20% Mini-PROTEAN TGX Precast Protein Gels. Proteins were transferred to polyvinylidene fluoride membranes using a Transblot Turbo blotting system (Bio-Rad). Alternatively, protein lysates were analyzed by a capillary-based immunoassay system (JESS Simple Western system, ProteinSimple). Antibodies used were anti-FDX2 antiserum (18), anti-SDHB, anti-FDX1, and anti-ERCC2 (ProteinTech), anti-p53, anti-pS15-p53, anti-CHK1, anti-pS345-CHK1, anti-CHK2, anti-pT68-CHK2, anti-DNA-PKcs, anti-Cleaved PARP, and anti-pS2056-DNA-PKcs (CST), anti-PARP (abcam), anti-p21 (SantaCruz, Dallas, TX), anti-γH2AX (Millipore), anti-Actin (Merck), anti-HSP60 (Protein Simple), and anti-tubulin-alpha (MBL). Second antibodies used were anti-mouse IgG-horseradish peroxidase (HRP) and anti-rabbit IgG-HRP for Western blotting. Anti-mouse Ig-HRP and anti-rabbit Ig-HRP (all purchased from Protein Simple) were used for the JESS system. Antibodies were validated based on the size of band in Western blotting/JESS (molecular weight), specificity/selectivity assessed by using samples from knockdown/knockout/overexpressing/inhibitor-treated cells, and reproducibility of the results.

### Measurement of ROS and free Fe^2+^

FDX2-iKO cells precultured 3 days with or without Doxy were seeded into 96-well plates. Two days later, cells were stained with either MitoSOX Red or CellROX Green for ROS measurements in phenol red–free medium according to the manufacturer’s (Thermo Fisher Scientific) instructions. For free Fe^2+^ measurements, cells were stained with FerroOrange or Mito-FerroGreen. Cells on replicate plates were fixed in formaldehyde and stained by 4′,6-diamidino-2-phenylindole-stained for cell normalization. All fluorescent signals were measured using a Synergy H1 plate reader (BioTek).

### RNA-seq and data analysis

Total RNAs were prepared from FDX2-iKO JHOC5 cells cultured 5 days with or without Doxy. RNA-seq analysis was performed using NEBNext Poly(A) mRNA Magnetic Isolation Module (NEB) and a NEBNext Ultra II Directional RNA Library Prep Kit for Illumina (NEB).

### RNA interference

ON-target plus SMARTpool siRNA consisting of a mixture of four siRNAs designed to silence human *FDX2* (#112812: 5′-GAGCUGCAAUAAAUCGAUA-3′, 5′-GGC-CCAGAUUGAGGGAAUA-3′, 5′-CCGAGGAGAGGGAAGACGA-3′, 5′-GGUUUGAGUA-GGAGUGGAC-3′) was obtained from Horizon Discovery. An ON-target plus nontargeting control pool (Horizon discovery) served as control siRNA. siRNAs were transfected using LipofectAMINE RNAiMAX (Thermo Fisher Scientific) according to the manufacturer’s recommendation.

### Quantitative reverse-transcription polymerase chain reaction analyses

Total RNA was reverse-transcribed using random primers and Superscript III RTase (Thermo Fisher Scientific). Quantitative reverse-transcription polymerase chain reaction analyses were performed using a LightCycler 480 (Roche). LightCycler 480 probes master and TaqMan probes from a universal probe library (Roche) were used for analyses of *PBGD* (#25) and *IL1A* (#35) (numbers in parentheses indicate probe number). Primers used are listed in Table S3. Analyses of *GDF15*, *IGFBP1*, *CXCL16*, *IL1B*, *SERPINE1*, *IL6*, *CCL20*, and *IL18* were performed using a TaqMan Gene Expression Assay kit (Thermo Fisher Scientific), as indicated in Table S3.

### Proteome analysis

Protein samples were prepared from FDX2-iKO JHOC5 cells cultured 5 or 6 days with or without Doxy. Proteome analysis based on the LC-MS/MS method was performed using the services of Kazusa DNA Research Institute. Briefly, raw LC-MS/MS data was searched against an *in silico* predicted spectral library using DIA-NN.3 (version:1.8.1, https://github.com/vdemichev/DiaNN). Detailed information about LC-MS/MS analysis and data processing will be provided upon request.

### Metabolome analysis

FDX2-iKO JHOC5 cells were cultured 5 days with or without Doxy and collected. Levels of cellular metabolites were determined by metabolome analysis as described ref. (11). Energy charge was calculated using the following formulation: energy charge = ([XTP] + 0.5 × [XDP])/([XTP] + [XDP] + [XMP]).

### Comet assay

FDX2-iKO JHOC5 cells were cultured 6 days in presence or absence of Doxy, collected in Banbanker solution (Nippon Genetics), and frozen at −80 °C until use. Comet assays in alkaline conditions were performed using the Trevigen’s Comet Assay kit (Trevigen) as described (49). After electrophoresis, comet slides were fixed and stained with SYBR Gold (Thermo Fisher Scientific). Fluorescent images were obtained by BZ-X800 microscopy (KEYENCE). Comet tail moments were scored using CometScore 2.0 software (http://rexhoover.com/index.php?id=cometscore).

### SA-β-galactosidase assay

FDX2-iKO JHOC5 cells were cultured 7 days in the presence or absence Doxy and then fixed and stained using a senescence β-galactosidase staining kit (CST).

### CRISPR-KO of *TP53* gene

sgRNA-targeting human *TP53* gene (True Guide Synthetic sgRNA (hTP53), A35533) was purchased from Thermo Fisher Scientific. FDX2-iKO JHOC5 clone #1 cells were transfected with RNPs consisting of Cas9 protein and *TP53* sgRNA, as described above. p53-deficient cells were selected in 10 μM Nutlin-3 (Cayman) as described (31).

### Cell viability assay

Cells cultured in 96-well plates were stained with Cytotox Red (Satorius) to detect dead/dying cells. After obtaining Cytotox Red images, dying/dead cells were washed away with PBS, and remaining cells were fixed in formaldehyde and stained with SYTOX Green (Thermo Fisher Scientific) for counting. Image acquisition and analyses were done using the IncuCyte analyzer.

### Lipid peroxidation assay

Lipid peroxidation was assessed using a BODIPY-C11 fluorescent probe. FDX2-iKO cells were cultured 4 days in the presence or absence of Doxy, treated 2 h with ML-162 and iFSP1, and then incubated with 5 uM BODYPI-C11 for an additional 15 min. Fluorescent BODIPY-C11 signals were detected using a SA3800 flow cytometer (Sony) at FITC detection settings to quantify oxidized BODIPY C11.

### Annexin V and TUNEL assays

Annexin V binding was assessed using an annexin V-FITC apoptosis detection kit (Nacala). TUNEL assays were performed using a MEBSTAIN Apoptosis TUNEL Kit Direct (MBL). In both assays, cells were stained according to the manufacturer’s instructions. Fluorescent signals were detected using a SA3800 flow cytometer at FITC detection settings.

### Cell drug treatments

FDX2-iKO cells were pre-cultured 3 days in the presence or absence of Doxy and reseeded into 96-well plates. The next day, the GPX4 inhibitor ML-162, the FSP1 inhibitor iFSP1, and/or Fer-1 were added to the medium at indicated concentrations. The number of viable cells was determined on day 5. Doxorubicin treatment was performed in 6 cm dishes after preculturing cells 4 days with or without Doxy. Cells were treated 20 h before preparation of cell lysates for Western blotting. For caspase inhibition experiments, cells were reseeded into 96-well plates after 4 days of preculture with or without Doxy. Then, on day 5, cells were treated with the pan-caspase inhibitor Z-VAD-FMK at 20 μM, and the number of cells was determined on day 8.

### Statistical analysis

No statistical methods were used to predetermine sample size. Experiments were not randomized nor were investigators blinded to allocation during experiments and outcome assessment. Student’s *t* test (two-tailed) and a one-way ANOVA followed by a Turkey’s post hoc test were used when comparing two groups and multiple groups, respectively. A *p* value of < 0.05 was considered significant. Data are presented as mean with the range or SEM.

## Data availability

Metabolome data are available at Metabolomics Workbench (https://www.metabolomicsworkbench.org/) (50) with the dataset identifier PR002029. Proteome data have been deposited to the JPOST (https://repository.jpostdb.org/) with the dataset identifier JPST003271. RNA-seq data have been deposited to the DDBJ (https://ddbj.nig.ac.jp/search) with the dataset identifier PRJDB18631. Data analyzed in Fig. S1*A* were obtained from cBioPortal at http://www.cbioportal.org/. Data analyzed in Fig. S1, *B–E* were obtained from DepMap at https://depmap.org/portal/download/. Source data are provided with this paper as a Source Data file. All unique materials used in this study are available upon request.

## Supporting information

This article contains supporting information.

## Conflict of interest

The authors declare that they have no conflicts of interest with the contents of this article.
