# Supplementary material for: FDX2, an iron-sulfur cluster assembly factor, is essential to prevent cellular senescence, apoptosis or ferroptosis of ovarian cancer cells
Source: J Biol Chem. 2024 Aug 14;300(9):107678. doi: 10.1016/j.jbc.2024.107678 (PMC11414659; doi:10.1016/j.jbc.2024.107678)
Supplement: Supplemental Figures S1–S6 and Tables S1–S3 [file mmc1.pdf]

Supporting information

FDX2, an iron-sulfur cluster assembly factor, is essential to prevent cellular senescence, apoptosis or ferroptosis of ovarian cancer cells.

Shuko Miya-hara, Mai Ohuchi, Miyuki Nomura, Eifumi Hashimoto, Tomoyoshi Soga, Rintaro Saito, Kayoko Hayashi, Taku Sato, Masatoshi Saito, Yoji Yamashita, Muneaki Shimada, Nobuo Yaegashi, Hidekazu Yamada, Nobuhiro Tanuma

- Figure S1, related to Fig. 1
- Figure S2, related to Fig. 1
- Figure S3, related to Fig. 2
- Figure S4, related to Fig. 3
- Figure S5, related to Fig. 4
- Figure S6
- Table S1, related to Fig. 2
- Table S2, related to Fig. 2
- Table S3, related to Fig. S4

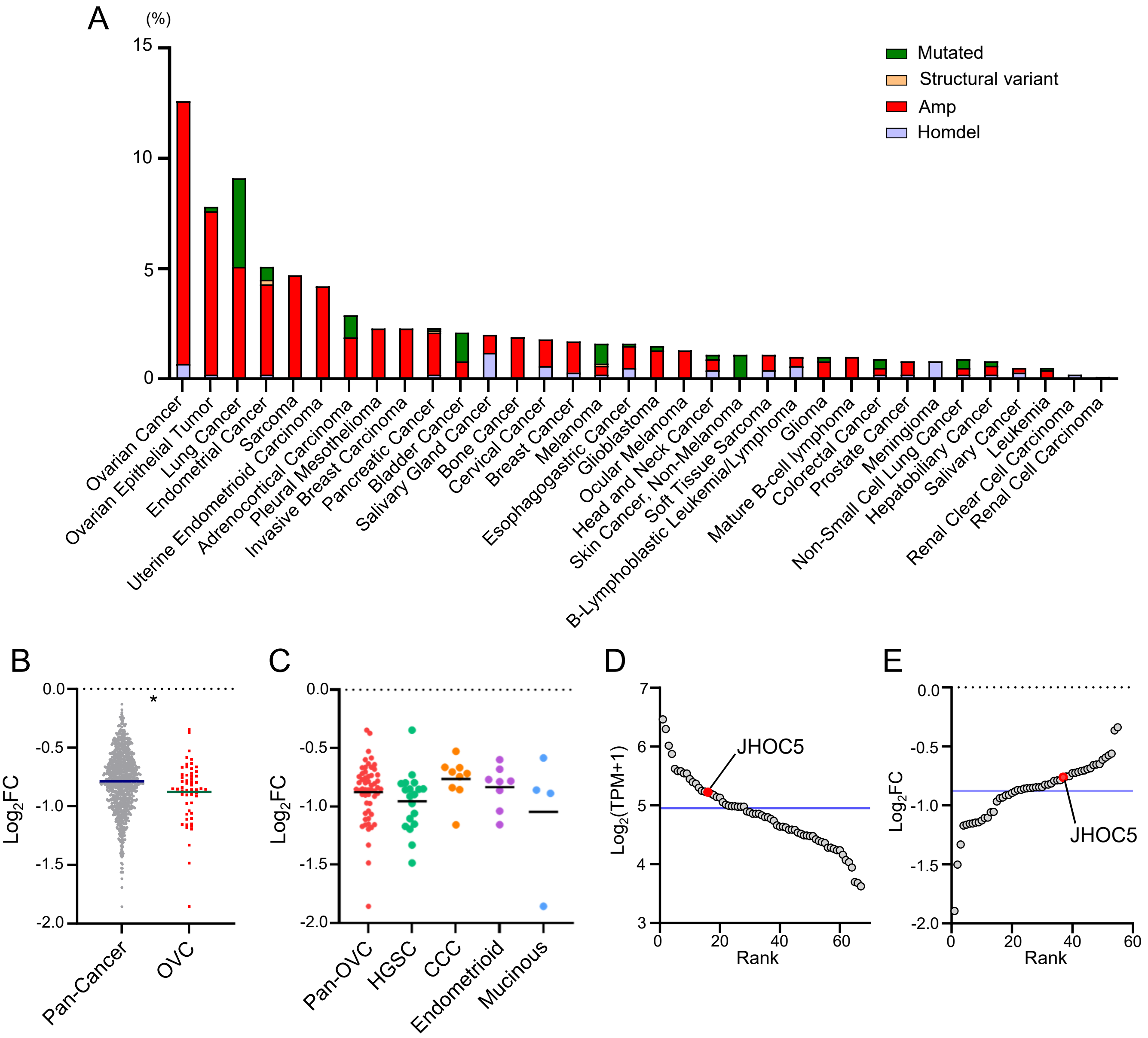

Figure S1. *FDX2* analysis using TCGA and DepMap datasets, related to Fig. 1.

(**A**) *FDX2* gene alteration frequency in human cancers collected in TCGA cohorts. Graph show sonly cohorts in which at least one gene alteration event is reported. Amp, amplification. Homdel, homozygous deletion.

(**B**) Analysis of the DepMap dataset showing effects of *FDX2*-KO on proliferation of cell lines in the CCLE collection. Shown are results in pan-cancer versus OVC categories. Symbols represent individual lines. n = 1070 and 55 for Pan-cancer and OVC groups, respectively.

(**C**) Further assessment of effects of *FDX2*-KO in ovarian cancer groups shown in B among histological subtypes. Symbols represent individual lines. n = 55, 19, 9, 8 and 4 for Pan-OVC, HGSC, CCC, Endometrioid and Mucinous groups, respectively. HGSC, high-grade serous carcinoma. CCC, clear cell carcinoma.

(**D**) Rank plot showing *FDX2* transcript levels in 67 OVC lines in the CCLE collection. Symbols represent individual lines. Blue line at 4.96 represents the average of 67 lines. JHOC5 is highlighted in red.

(**E**) Rank plot showing *FDX2*-KO effects on proliferation of 55 OVC lines shown in B. Symbols represent individual lines. Blue line at 0.878 represents the average of 55 lines. JHOC5 is highlighted in red.

Bars represent mean (B, C). $^*p < 0.05$ as determined by two-tailed t-test. Source data and exact p values are provided as a Source Data file.

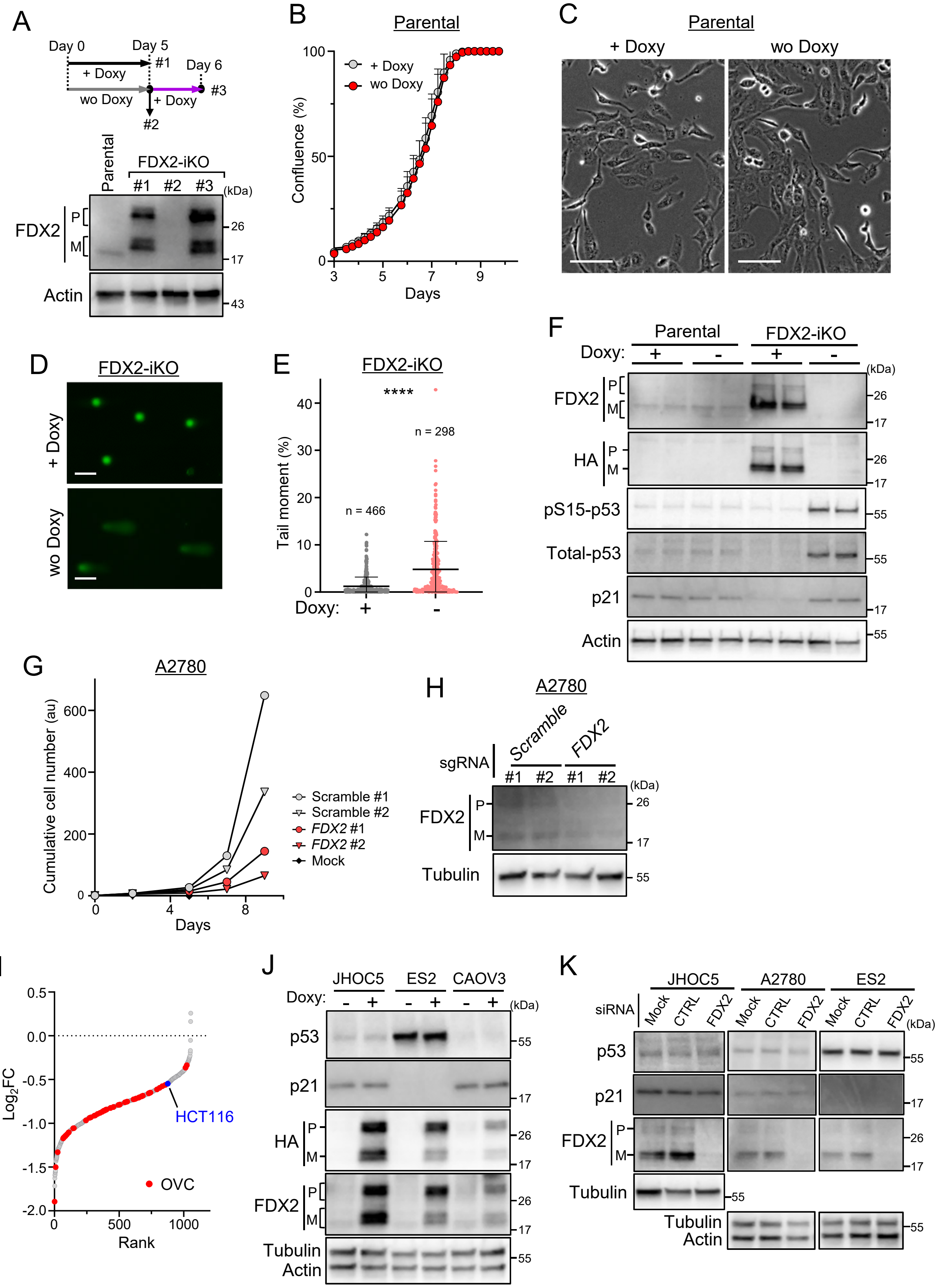
A
Day 0
Day 5
#1
Day 6
+ Doxy
#3
wo Doxy
+ Doxy
#2
Parental
FDX2-iKO
#1 #2 #3
(kDa)
FDX2
P
M
26
17
Actin
43
B
Parental
100
+ Doxy
wo Doxy
Confluence (%)
50
0
3 5 7 9
Days
C
Parental
+ Doxy
wo Doxy
D
FDX2-iKO
+ Doxy
wo Doxy
E
FDX2-iKO
****
40
n = 298
30
Tail moment (%)
20
n = 466
10
0
Doxy: + -
F
Parental
FDX2-iKO
Doxy: + - + -
(kDa)
FDX2
P
M
26
17
HA
P
M
26
17
pS15-p53
55
Total-p53
55
p21
17
Actin
55
G
A2780
600
400
Cumulative cell number (au)
200
0
0 4 8
Days
Scramble #1
Scramble #2
FDX2 #1
FDX2 #2
Mock
H
A2780
Scramble
FDX2
sgRNA
#1 #2 #1 #2
(kDa)
FDX2
P
M
26
17
Tubulin
55
I
0.5
0.0
-0.5
Log2FC
-1.0
-1.5
-2.0
HCT116
OVC
0 500 1000
Rank
J
JHOC5 ES2 CAOV3
Doxy: - + - + - +
(kDa)
p53
55
p21
17
HA
P
M
26
17
FDX2
P
M
26
17
Tubulin
Actin
55
K
JHOC5 A2780 ES2
siRNA
Mock CTRL FDX2 Mock CTRL FDX2 Mock CTRL FDX2
(kDa)
p53
55
p21
17
FDX2
P
M
26
17
Tubulin
55
Tubulin
Actin
55

Figure S2. Effects of FDX2 loss in p53-proficient OVC cells, related to Fig. 1.

(**A**) Western blot showing FDX2 protein expression in FDX2-iKO JHOC5 cells following Doxy-withdrawal and Doxy-re-feeding. P and M denote pre- and mature forms of FDX2, respectively.

(**B**) Proliferation of parental JHOC5 cells cultured with or without Dox. Shown are representative results of experiments repeated twice.

(**C**) Representative phase contrast images of parental JHOC5 cells cultured 7 days with or without Doxy. Scale bars, 100 μm.

(**D**) Representative images of results of comet assays of FDX2-iKO JHOC5 cells cultured with or without Doxy for 6 days. Scale bars, 100 μm.

(**E**) Quantification of results (tail moments) shown in D. Shown are representative results of two experiments.

(**F**) Parental JHOC5 and FDX2-iKO JHOC5 cells were cultured 6 days with or without Doxy, and assayed for Ser15-phosphorylated (pS15) p53, total p53 and p21 protein levels by Western blotting. In the FDX2 and HA blot, P and M are as in A. Note that some of these results are shown also in Fig. 1H.

(**G**) Proliferation of A2780 cells transduced with Cas9 plus either *FDX2* sgRNAs or control sgRNAs or mock-infected (Mock). Shown are representative results of experiments repeated twice.

(**H**) Western blotting of cells indicated in G. In the FDX2 blot, P and M are as in A.

(**I**) Rank plot of *FDX2*-KO effects on proliferation of cell lines in the CCLE collection. Symbols represent individual lines. OVC lines and HCT116 are highlighted in red and blue, respectively.

(**J**) Western blot showing levels of p53 and p21 before and after FDX2 overexpression in OVC cells. Cells expressing FDX2/HA Doxy-dependently were cultured 3 days in the presence or absence of Doxy, and analyzed. P and M in the FDX2 and HA blots are as in B.

(**K**) Western blot showing levels of p53 and p21 before and after FDX2 knockdown in OVC cells. Cells were transfected with indicated siRNAs or mock-transfected, cultured 3 days, and analyzed. P and M in the FDX2 blot are as in B. Note that p21 is under the detection limit in ES2 cells.

Data are presented as mean plus SD (B) or SEM (E). ****$p < 0.0001$ as determined by two-tailed t-test (**E**) Source data and the exact *p* values are provided as a Source Data file.

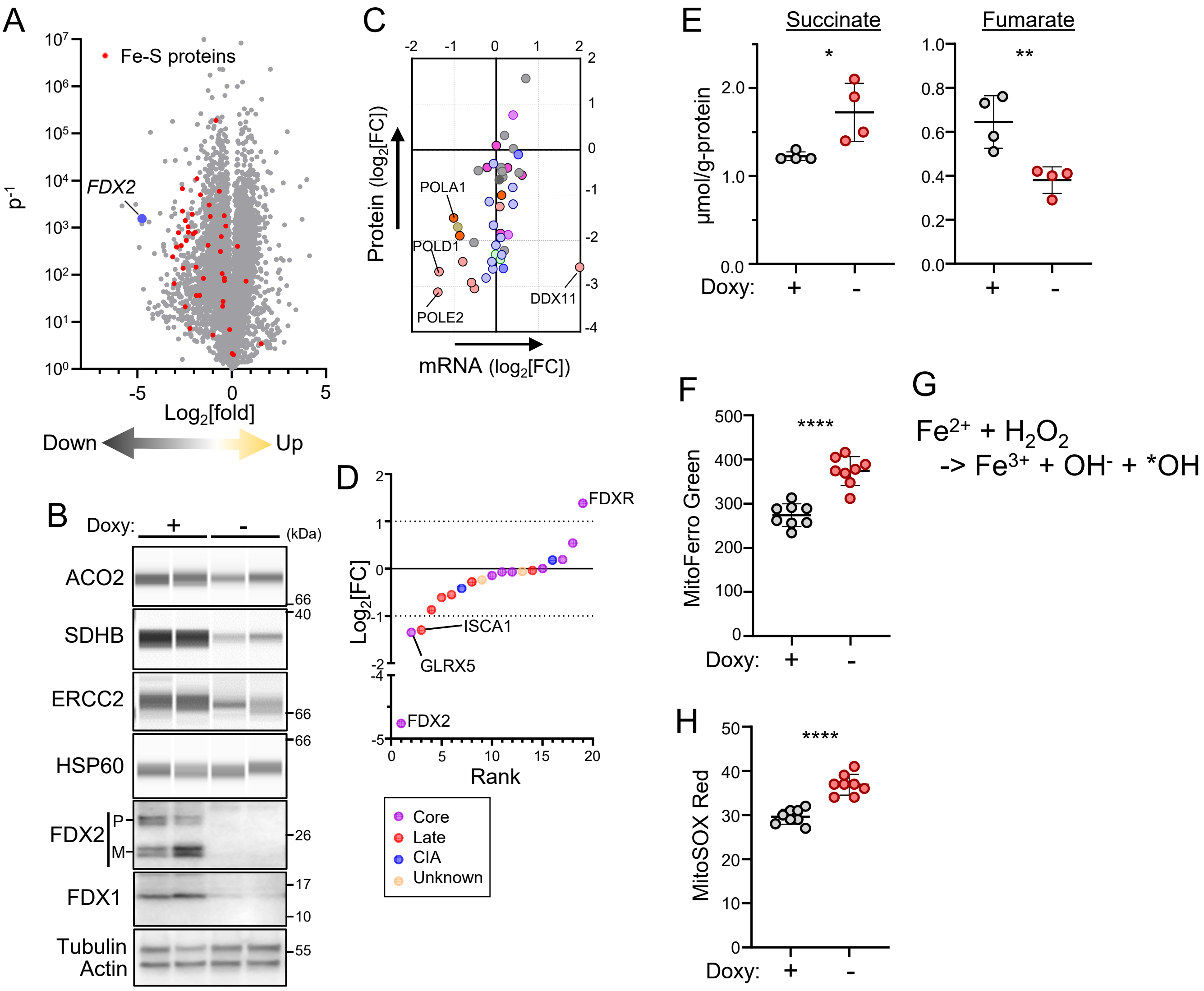

Figure S3. Additional phenotypic studies of FDX2-iKO JHOC5 cells, related to Fig. 2.
(**A**) Volcano plot showing proteome changes following induction of FDX2 loss. Fe-S proteins and FDX2 itself are indicated in red and blue, respectively. *p* values were based on a two-tailed t-test.
(**B**) Immunoassays and Western blot analysis of 4 Fe-S proteins (ACO2, SDHB, ERCC2 and FDX1) in FDX2-iKO cells, treated with or without Doxy. HSP60, Tubulin and Actin served as loading controls.
(**C**) 2D plot showing relationship between FDX2 depletion-induced changes in levels of proteins or mRNAs encoding Fe-S proteins shown in Fig. 2C. Symbol colors correspond to the classification system shown in Fig. 2C.
(**D**) Rank plot showing fold-change in levels of proteins functioning in either Fe-S assembly or Fe-S protein assembly, before and after FDX2 loss. Core, core Fe-S assembly machinery. Late, late Fe-S assembly machinery. CIA, cytosolic Fe-S assembly.
(**E**) Succinate (left) and fumarate (right) levels in FDX2-iKO JHOC5 cells cultured 5 days in the presence or absence of Doxy. n = 4 biological replicates.
(**F**) Mitochondrial free Fe^{2+} levels in FDX2-iKO cells cultured 5 days in the presence or absence of Doxy. n = 8 biological replicates.
(**G**) Fenton reaction, in which Fe^{2+} reacts with H_2O_2 to generate hydroxyl radicals.
(**H**) Mitochondrial ROS levels in FDX2-iKO cells cultured 5 days in the presence or absence of Doxy. n = 8 biological replicates.
Data are presented as mean plus SEM (E, F, H). *$p < 0.05$, **$p < 0.01$, ****$p < 0.0001$ as determined by two-tailed t-test (E, F, H). Source data and exact p values are provided as a Source Data file.

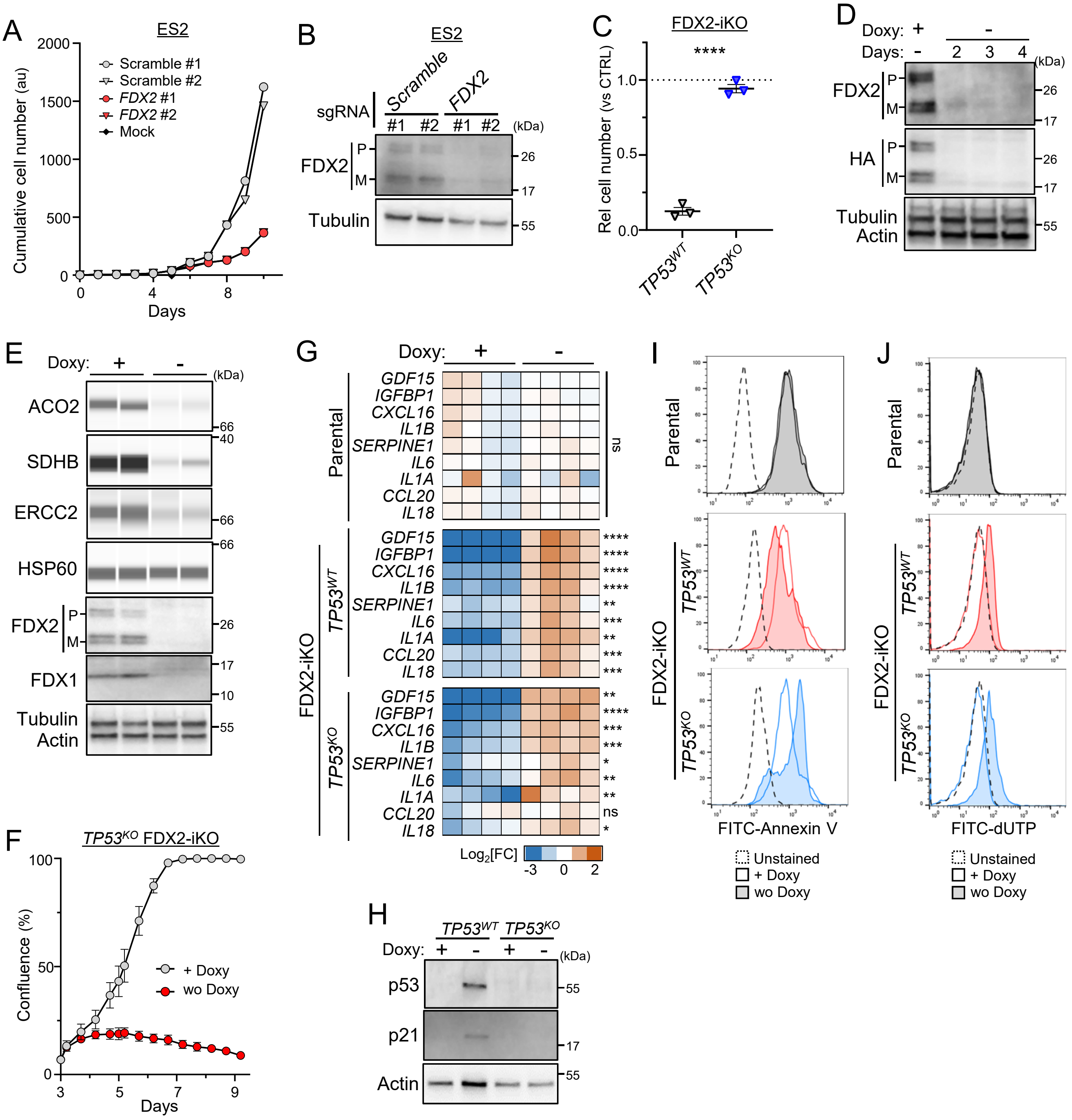

Figure S4. FDX2-KO effects in p53-deficient OVC cells, related to Fig. 3.
(**A**) Proliferation of ES2 cells transduced with Cas9 plus either *FDX2* sgRNAs or control sgRNAs or mock-infected (Mock). Shown are representative results of experiments repeated 3 times.
(**B**) Western blotting of cells indicated in A. In the FDX2 blot, P and M denote pre- and mature forms of FDX2, respectively.
(**C**) Nutlin-3 sensitivity of $TP53^{WT}$ and $TP53^{KO}$ FDX2-iKO JHOC5 cells. Cells were cultured 4 days in the presence of Doxy, with or without 10 μM nutlin-3. Shown are relative numbers of cells treated with nutlin-3. Values seen in cells not treated with nutlin-3 were arbitrarily defined as 1.0 (dashed line) in each genotype.

(**D**) Western blot showing time course of FDX2 protein loss after Doxy removal in *TP53*^{KO} FDX2-iKO JHOC5 cells. Tubulin serves as a loading control.
(**E**) Immunoassays and Western blot analysis showing levels of 4 Fe-S proteins (ACO2, SDHB, ERCC2 and FDX1) in *TP53*^{KO} FDX2-iKO cells grown 5 days with or without Doxy. HSP60, Tubulin and Actin served as loading controls. P and M in the FDX2 and HA blots are as in B.
(**F**) Proliferation of *TP53*^{KO} FDX2-iKO cells cultured with or without Doxy. Shown are representative results of experiments repeated twice.
(**G**) Expression of mRNA encoding indicated SASP factors in parental JHOC5, *TP53*^{WT} FDX2 iKO and *TP53*^{KO} FDX2-iKO cells. Cells were cultured 5 days in the presence or absence of Doxy before qRT-PCR analysis. n = 4 biological replicates.
(**H**) Western blot analyses of *TP53*^{WT} and *TP53*^{KO} FDX2-iKO cells with or without 6 days of Doxy treatment. Actin serves as a loading control.
(**I**) Analysis of annexin V binding activity of parental JHOC5, *TP53*^{WT} FDX2-iKO and *TP53*^{KO} FDX2-iKO cells. Cells were cultured 7 days with or without Doxy, and incubated with FITC-Annexin V. Fluorescent signals were detected by flowcytometry. Analysis shown is representative of 2-3 independent experiments.
(**J**) TUNEL assay of parental JHOC5, *TP53*^{WT} FDX2-iKO and *TP53*^{KO} FDX2-iKO cells. Cells were cultured 7 days with or without Doxy, and subjected to TUNEL staining. Fluorescent signals of FITC-dUTP were detected by flowcytometry. Cells stained without TdT enzyme served as unstained controls. Shown are representatives of independent experiments repeated twice.
Data are presented as mean plus SEM (C) or SD (F). *$p < 0.05$, **$p < 0.01$, ***$p < 0.001$, ****$p < 0.0001$ as determined by two-tailed t-test (C and G). Source data and the exact *p* values are provided as a Source Data file.

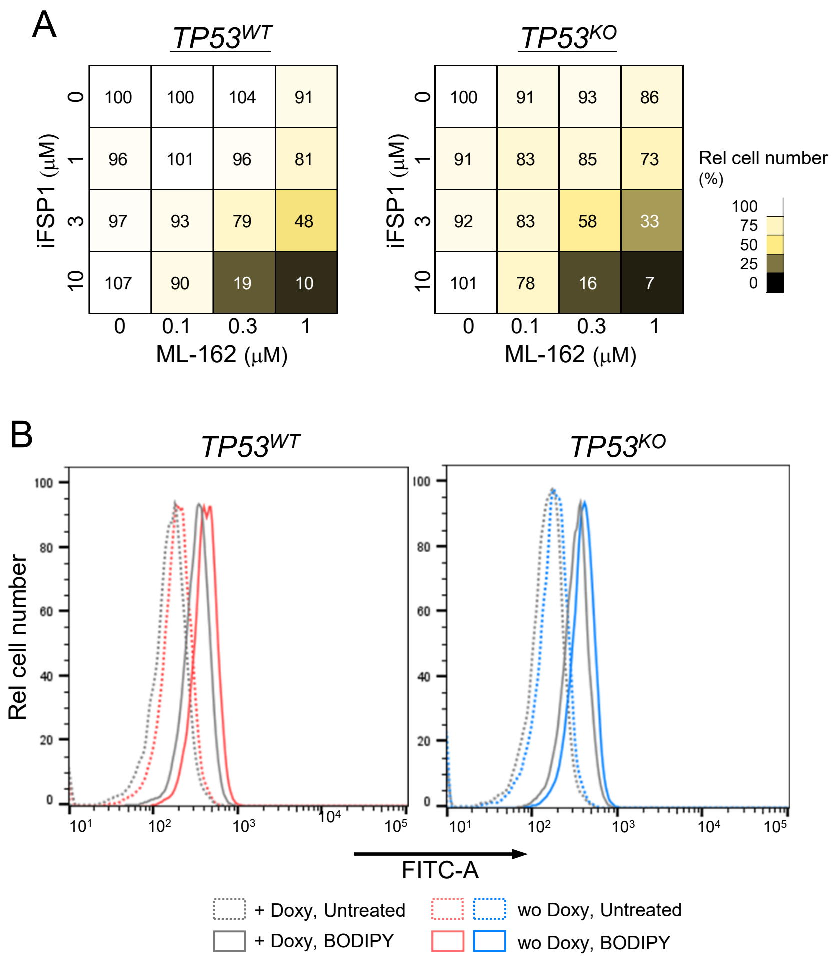

Figure S5. Analysis of ferroptosis of FDX2-iKO JHOC5 cells, related to Fig. 4
(**A**) Effects of GPX4/FSP1 dual inhibition on cell numbers. FDX2-iKO cells (+ Doxy culture) were treated 1 day with ML-162 and iFSP1 at indicated concentrations. The number of viable cells was determined by SRB staining.
(**B**) Comparison of BODIPY C11-stained or unstained FDX2-iKO JHOC5 cells. Cells were cultured with or without Doxy for 4 days, stained with BODIPY C11 or left unstained, and analyzed by flowcytometry.
Data are presented as mean values (A). Source data are provided as a Source Data file.

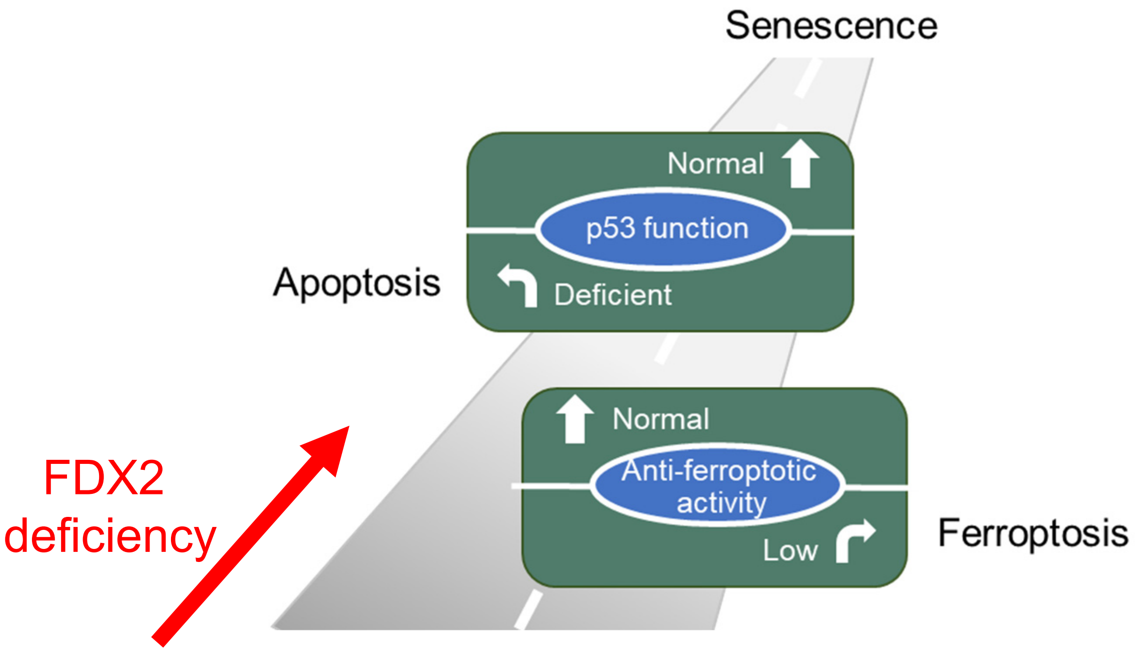

Figure S6. Cell fate decisions of cancer cells after FDX2-deficiency.

Table S1. Rel abundance of Fe-S proteins in FDX2-iKO JHOC5 cells

Protein Name	Gene symbol	Class	Proteome analyses											RNAseq
			Z score							Log2[FC]	p-value	Sig. change	Rank	Log2[FC]
			+ Dox (1)	+ Dox (2)	+ Dox (3)	wo Dox (1)	wo Dox (2)	wo Dox (3)	wo Dox (4)					
DNA polymerase epsilon subunit 2	*POLE2*	DNA repair	1.058	0.974	1.002	-0.421	-0.512	-0.675	-1.426	-3.13	0.0042	Yes	1	-1.384
Adenine DNA glycosylase	*MUTYH*	DNA repair	0.967	0.952	0.913	-1.389	0.177	-0.652	-0.968	-3.05	0.0154	Yes	2	-0.517
Endonuclease III-like protein 1	*NTHL1*	DNA repair	0.942	1.098	1.020	-0.282	-0.596	-0.931	-1.251	-2.92	0.0026	Yes	3	-0.587
NADH dehydrogenase [ubiquinone] iron-sulfur protein 8, mitochondrial	*NDUFS8*	ETC/TCA	0.998	1.094	1.026	-0.486	-0.503	-1.097	-1.032	-2.82	0.0013	Yes	4	-0.241
DNA polymerase delta catalytic subunit	*POLD1*	DNA repair	0.999	1.031	1.057	-0.459	-0.423	-1.133	-1.071	-2.68	0.0024	Yes	5	-1.358
Succinate dehydrogenase [ubiquinone] iron-sulfur subunit, mitochondrial	*SDHB*	ETC/TCA	1.088	1.059	1.036	-0.604	-0.700	-0.929	-0.951	-2.62	0.0001	Yes	6	-0.079
Ferrochelatase, mitochondrial	*FECH*	Heme	1.048	1.048	1.071	-0.543	-0.676	-1.025	-0.923	-2.61	0.0004	Yes	7	0.171
ATP-dependent DNA helicase DDX11	*DDX11*	DNA repair	0.927	0.974	1.057	-0.246	-0.297	-1.128	-1.287	-2.58	0.0073	Yes	8	1.984
NADH dehydrogenase [ubiquinone] iron-sulfur protein 7, mitochondrial	*NDUFS7*	ETC/TCA	1.047	1.084	1.016	-0.554	-0.553	-0.999	-1.041	-2.46	0.0007	Yes	9	-0.101
DNA replication ATP-dependent helicase/nuclease DNA2	*DNA2*	DNA repair	0.769	0.826	0.884	0.014	-0.009	-1.898	-0.586	-2.46	0.0480	Yes	10	-0.792
Adrenodoxin, mitochondrial	*FDX1*	Lipoylation	1.046	1.055	1.003	-0.445	-0.495	-1.069	-1.095	-2.41	0.0019	Yes	11	0.102
NADH-ubiquinone oxidoreductase 75 kDa subunit, mitochondrial	*NDUFS1*	ETC/TCA	1.059	1.058	1.024	-0.502	-0.579	-1.013	-1.047	-2.31	0.0010	Yes	12	0.132
Lipoyl synthase, mitochondrial	*LIAS*	Lipoylation	0.979	1.109	1.022	-0.732	-0.361	-0.826	-1.191	-2.30	0.0012	Yes	13	-0.015
NADH dehydrogenase [ubiquinone] flavoprotein 2, mitochondrial	*NDUFV2*	ETC/TCA	1.111	0.998	1.037	-0.509	-0.608	-0.963	-1.066	-2.10	0.0005	Yes	15	-0.007
Amidophosphoribosyltransferase	*PPAT*	Nucleotide metab	0.886	0.986	1.183	-0.387	-0.430	-1.088	-1.151	-2.04	0.0014	Yes	16	-0.526
NADH dehydrogenase [ubiquinone] flavoprotein 1, mitochondrial	*NDUFV1*	ETC/TCA	1.059	1.034	1.037	-0.532	-0.502	-1.061	-1.035	-1.93	0.0013	Yes	17	0.109
DNA primase large subunit	*PRIM2*	DNA replication	0.950	0.934	1.076	-0.264	-0.279	-1.217	-1.200	-1.89	0.0069	Yes	18	-0.866
CDGSH iron-sulfur domain-containing protein 3, mitochondrial	*CISD3*	Iron	0.982	0.432	1.127	0.069	-1.687	-0.850	-0.072	-1.87	0.0277	Yes	19	0.285
Mitochondrial tRNA methylthiotransferase CDK5RAP1	*CDK5RAP1*	tRNA	1.124	1.036	1.016	-0.559	-0.718	-0.952	-0.946	-1.84	0.0001	Yes	20	0.103
Chromosome-associated kinesin KIF4A	*KIF4A*	Mitosis	0.876	0.908	0.898	0.027	-0.024	-1.324	-1.360	-1.70	0.0274	Yes	21	-0.913
Cytochrome b-c1 complex subunit Rieske, mitochondrial (RISP)	*UQCRFS1*	ETC/TCA	1.096	0.929	1.120	-0.520	-0.595	-1.031	-0.998	-1.66	0.0002	Yes	22	-0.065
DNA polymerase alpha catalytic subunit	*POLA1*	DNA replication	0.934	0.957	0.993	-0.166	-0.207	-1.234	-1.278	-1.50	0.0120	Yes	23	-1.012
General transcription and DNA repair factor IIH helicase subunit XPD	*ERCC2*	DNA repair	1.034	1.025	1.034	-0.535	-0.372	-1.076	-1.109	-1.25	0.0024	Yes	24	0.085
Electron transfer flavoprotein-ubiquinone oxidoreductase, mitochondrial	*ETFDH*	ETC/TCA	0.972	1.178	0.977	-0.489	-0.597	-1.154	-0.887	-1.20	0.0003	Yes	25	0.400
Succinate dehydrogenase [ubiquinone] flavoprotein subunit, mitochondrial	*SDHA*	ETC/TCA	1.085	0.963	1.088	-0.505	-0.563	-1.055	-1.014	-1.14	0.0006	Yes	26	-0.166
Regulator of telomere elongation helicase 1	*RTEL1*	DNA replication	0.653	0.690	0.360	-1.274	-0.451	-1.242	1.264	-1.00	0.1931	No	27	0.131
Aconitate hydratase, mitochondrial	*ACO2*	ETC/TCA	1.076	1.069	1.059	-0.720	-0.850	-0.796	-0.838	-0.83	0.0000	No	28	0.395
CDGSH iron-sulfur domain-containing protein 2	*CISD2*	Autophagy/ER	1.069	1.024	1.086	-0.582	-0.713	-0.893	-0.992	-0.66	0.0002	No	29	0.072
Elongator complex protein 3	*ELP3*	Transcription	0.686	0.897	1.341	-0.337	-0.238	-1.153	-1.195	-0.60	0.0032	No	30	0.160
Threonylcarbamoyladenosine tRNA methylthiotransferase	*CDKAL1*	tRNA	1.078	1.081	0.928	-0.326	-0.614	-0.971	-1.176	-0.56	0.0016	No	31	0.614
2-(3-amino-3-carboxypropyl)histidine synthase subunit 1	*DPH1*	Translation	0.548	0.995	1.480	-0.809	-0.414	-0.709	-1.091	-0.51	0.0095	No	32	0.543
2-(3-amino-3-carboxypropyl)histidine synthase subunit 2	*DPH2*	Translation	0.274	0.720	1.738	-0.313	-0.351	-1.148	-0.921	-0.48	0.0468	No	33	0.143
ATP-binding cassette sub-family E member 1	*ABCE1*	Translation	0.404	0.849	1.195	0.084	0.169	-1.405	-1.296	-0.46	0.0372	No	34	-0.426
S-adenosyl-L-methionine-dependent tRNA 4-demethylwyosine synthase TYW1	*TYW1*	tRNA	0.848	1.315	0.906	-0.457	-0.589	-0.760	-1.264	-0.40	0.0006	No	35	0.273
Cleavage and polyadenylation specificity factor subunit 4	*CPSF4*	Transcription	0.988	0.939	0.962	-0.233	-0.158	-1.345	-1.154	-0.39	0.0118	No	36	0.115
CDK5 regulatory subunit-associated protein 2	*CDK5RAP2*	tRNA	0.535	0.997	1.183	-0.145	-0.023	-1.108	-1.439	-0.39	0.0135	No	37	-0.215
CDGSH iron-sulfur domain-containing protein 1	*CISD1*	ETC/TCA	1.328	0.967	0.750	-0.304	-0.612	-1.007	-1.121	-0.31	0.0009	No	38	-0.066
Radical S-adenosyl methionine domain-containing protein 1, mitochondrial	*RSAD1*	Heme	0.016	0.592	1.253	-0.019	-1.968	0.379	-0.254	-0.11	0.1464	No	39	0.524
Cytoplasmic tRNA 2-thiolation protein 1	*CTU1*	tRNA	-1.650	-0.610	1.121	0.980	0.704	-0.421	-0.125	0.09	0.5084	No	41	0.003
Glutaredoxin-2, mitochondrial	*GLRX2*	Redox	-0.709	-0.897	-1.346	1.196	1.060	0.553	0.143	0.31	0.0025	No	42	0.198
Cytoplasmic aconitate hydratase	*ACO1*	Iron	-1.426	-1.112	-0.535	0.584	0.688	0.835	0.966	0.76	0.0137	No	43	0.402
Xanthine dehydrogenase/oxidase	*XDH*	Nucleotide metab	-2.186	0.327	-0.016	0.302	0.166	0.740	0.667	1.56	0.2977	No	44	0.706

Heme, heme biosynthesis. tRNA, tRNA synthesis and modification. Iron, iron regulation.

Table S2. Rel abundance of Fe-S/Fe-S-protein assembly proteins in FDX2-iKO JHOC5 cells

Protein Name	Gene Symbol	Class	Z score							Log_2[FC]	p-value	Sig. change	Rank
			+ Dox (1)	+ Dox (2)	+ Dox (3)	wo Dox (1)	wo Dox (2)	wo Dox (3)	wo Dox (4)				
Cytosolic iron-sulfur assembly component 3	*CIAO3*	CIA	0.527	0.985	1.500	-0.612	-0.913	-0.437	-1.050	-0.42	0.0113	No	7
Anamorsin	*CIAPIN1*	CIA	-1.848	-0.708	0.761	0.503	0.973	-0.196	0.515	0.18	0.2978	No	16
Ferredoxin-2, mitochondrial	*FDX2*	Core	1.056	1.022	1.075	-0.452	-1.023	-0.727	-0.951	-4.76	0.0006	Yes	1
Glutaredoxin-related protein 5, mitochondrial	*GLRX5*	Core	1.084	0.846	0.888	-0.037	-0.404	-0.779	-1.597	-1.35	0.0139	Yes	2
GrpE protein homolog 1, mitochondrial	*GRPEL1*	Core	1.222	0.730	0.820	0.219	-0.596	-1.067	-1.327	-0.15	0.0117	No	10
Cysteine desulfurase	*NFS1*	Core	1.394	-0.055	0.179	-1.227	-1.318	0.869	0.157	-0.07	0.2610	No	11
Frataxin, mitochondrial	*FXN*	Core	0.812	1.753	-0.279	-0.033	-0.185	-0.936	-1.132	-0.07	0.1357	No	12
Stress-70 protein, mitochondrial	*HSPA9*	Core	0.462	0.318	-0.816	0.483	-1.905	0.947	0.512	0.00	0.9788	No	15
Iron-sulfur cluster assembly enzyme ISCU	*ISCU*	Core	0.746	-0.836	-1.580	1.111	-0.520	0.824	0.255	0.19	0.2950	No	17
GrpE protein homolog 2, mitochondrial	*GRPEL2*	Core	-0.854	-1.141	-1.177	0.846	0.704	0.977	0.646	0.54	0.0001	No	18
NADPH:adrenodoxin oxidoreductase, mitochondrial	*FDXR*	Core	-0.992	-1.077	-1.083	0.556	0.577	1.058	0.960	1.38	0.0005	Yes	19
Iron-sulfur cluster assembly 1 homolog, mitochondrial	*ISCA1*	Late	1.164	0.990	1.020	-0.511	-0.890	-0.865	-0.908	-1.30	0.0000	Yes	3
NFU1 iron-sulfur cluster scaffold homolog, mitochondrial	*NFU1*	Late	1.028	1.073	1.074	-0.591	-0.678	-0.873	-1.033	-0.87	0.0003	No	4
Iron-sulfur protein NUBPL	*NUBPL*	Late	1.541	0.814	0.745	-0.800	-0.756	-0.807	-0.738	-0.61	0.0189	No	5
Iron-sulfur cluster assembly 2 homolog, mitochondrial	*ISCA2*	Late	1.166	1.003	1.014	-0.941	-0.803	-0.863	-0.575	-0.55	0.0000	No	6
Putative transferase CAF17, mitochondrial	*IBA57*	Late	0.532	1.331	1.222	-0.780	-0.749	-0.968	-0.589	-0.28	0.0126	No	8
BolA-like protein 3	*BOLA3*	Late	2.035	-0.285	-1.207	0.203	-0.041	-0.234	-0.471	-0.04	0.7749	No	14
Iron-sulfur cluster co-chaperone protein HscB	*HSCB*	Unknown	0.965	0.870	1.231	-0.453	-0.541	-1.313	-0.759	-0.24	0.0008	No	9
LYR motif-containing protein 4	*LYRM4*	Unknown	1.869	-0.478	0.054	-0.542	-1.361	0.123	0.336	-0.06	0.3695	No	13

Core, core Fe-S assembly machinery. Late, late Fe-S assembly machinery. CIA, cytosolic Fe-S assembly.

Table S3. qRT-PCR analysis

Assays using UPL (Roche)			
Target	***Sense***	***Anti-sense***	***Probe***
IL1A	5'-aaccagtgctgctgaagga-3'	5'-ttcttagtgccgtgagtttcc-3'	UPL #35
PBGD	5'-agctatgaaggatgggcaac-3'	5'-ttgtatgctatctgagccgtcta-3'	UPL #25

Assays using TaqMan Gene Expression Assay (Thermo Fisher)	
Target	***Identifier***
GDF15	Hs00171132_m1
IGFBP1	Hs00236877_m1
CXCL16	Hs00222859_m1
IL1B	Hs01555410_m1
SERPINE1	Hs00167155_m1
IL6	Hs00174131_m1
CCL20	Hs00355476_m1
IL1B	Hs01555410_m1
